# Repurposing Insecticides for Mosquito Control: Evaluating Spiromesifen, a Lipid Synthesis Inhibitor against *Aedes aegypti* (L.)

**DOI:** 10.3390/tropicalmed9080184

**Published:** 2024-08-18

**Authors:** Daniela Cerda-Apresa, Selene M. Gutierrez-Rodriguez, Jesus A. Davila-Barboza, Beatriz Lopez-Monroy, Iram P. Rodriguez-Sanchez, Karla L. Saavedra-Rodriguez, Adriana E. Flores

**Affiliations:** 1 Facultad de Ciencias Biologicas, Universidad Autonoma de Nuevo Leon, Av. Universidad s/n Cd. Universitaria, San Nicolas de los Garza 66455, NL, Mexico; daniela.cerdaap@uanl.edu.mx (D.C.-A.); selene.gutierrezro@uanl.edu.mx (S.M.G.-R.); jdavilab@uanl.edu.mx (J.A.D.-B.); beatriz.lopezmr@uanl.edu.mx (B.L.-M.); iram.rodriguezsa@uanl.edu.mx (I.P.R.-S.); 2 Department of Microbiology, Immunology and Pathology, Colorado State University, Fort Collins, CO 80523, USA; karla.saavedra_rodriguez@colostate.edu

**Keywords:** *Aedes aegypti*, insecticide resistance, lipid synthesis inhibition, spiromesifen, sterilizing effect, vector control

## Abstract

The growing resistance of *Aedes aegypti* (L.) to conventional insecticides presents a major challenge in arbovirus control, necessitating the exploration of alternative insecticidal chemistries. Spiromesifen, derived from spirocyclic tetronic acids, is widely used against agricultural pests and is crucial in resistance management due to its unique lipid synthesis inhibition. This study evaluates the insecticidal activity of spiromesifen against temephos-resistant *Ae. aegypti* populations, focusing on larval body weight, volume, biochemical composition, and adult female reproductive potential. Spiromesifen demonstrated effective larvicidal activity, significantly reducing adult emergence. Resistance to spiromesifen was not observed, with resistance ratios (RR_50_, RR_90_) ranging from 0.36- to 3.31-fold. Larvae exposed to LC_50_ showed significant reductions in body weight and volume, and reduced carbohydrate, lipid, and protein contents. Enhanced catalase activity and malondialdehyde levels indicated increased oxidative stress and lipid peroxidation, highlighting its effects on lipid metabolism. Spiromesifen also exhibited sterilizing effects, significantly reducing fecundity and fertility in adult females, thereby impacting *Ae. aegypti* reproductive capacity. These findings highlight the potential of spiromesifen as a component of integrated vector management strategies, especially in regions with prevalent insecticide resistance in *Ae. aegypti*, serving as an effective larvicide and impacting adult reproductive outcomes.

## 1. Introduction

*Aedes aegypti* (L.), the primary vector for arboviruses, including dengue, Zika, chikungunya, and yellow fever, is widely distributed in tropical and subtropical regions, placing approximately 2.5 billion people at risk [1]. Vector control remains the primary strategy for disease management [2,3].

Mosquito control has predominantly relied on neurotoxic chemical insecticides, which target the nervous system of mosquitoes, leading to paralysis and death [4,5,6,7]. Since the 1950s, vector control programs in Mexico have employed various classes of chemical insecticides [8]. Temephos, a larvicide used for over 50 years, remains the most widely used for larval control [9,10]. However, its effectiveness has declined due to the increasing resistance in target populations, a global phenomenon exacerbated by ongoing insecticide pressure [11]. A recent study has confirmed widespread resistance to temephos in *Ae. aegypti* populations across Mexico [10].

The same phenomenon occurs with adulticides; although organophosphates and carbamates are authorized for use in Mexico, pyrethroids have been the most widely used insecticides since 1999 [8,12], leading to widespread resistance in *Ae. aegypti* populations [12,13,14,15,16,17,18,19,20,21,22,23].

Insecticide resistance is a significant barrier to effective vector control, and as a result, control programs must continuously adapt by switching to different insecticides. However, there is currently a notable deficiency in alternative insecticides that are both cost-effective and safe, making the development of new insecticides a critical priority. Nevertheless, this task is marked by high costs and the necessity for long-term research commitments; despite the undeniable utility of insecticides in reducing pathogen transmission, few new insecticides are specifically developed and marketed for vector control due to the high costs and low profitability in the vector control market [24].

The global agrochemical market for crop protection was valued at USD 61.42 billion in 2023 and is projected to grow to USD 64.18 billion in 2024, while the market for insecticides, specifically for vector control, is much smaller, valued at up to USD 500 million at the active ingredient level [25,26]. This segment represents a minor part of the non-crop agrochemical market, reflecting its specialized nature and limited scale compared to crop protection products [27]. The high costs of developing new active ingredients over the last two decades, driven by extensive research, development, and regulatory hurdles, have negatively impacted interest in minor markets such as vector control [25].

Using existing insecticides initially developed for purposes unrelated to vector control presents an alternative strategy for managing resistance associated with conventional insecticides [28]. This approach is being explored by groups such as the Innovative Vector Control Consortium (IVCC), which evaluates insecticidal products used in crop protection and animal health for their efficacy against mosquitoes, particularly *Anopheles gambiae* Giles [29]. By repurposing these existing products, it is possible to leverage already established safety and efficacy profiles, potentially accelerating the availability of effective vector control solutions and reducing development costs.

Spiromesifen, an insecticide/acaricide derived from spirocyclic tetronic acid, is classified in Group 23 by the Insecticide Resistance Action Committee (IRAC) based on its mode of action [30]. This insecticide, synthesized in 1994 and commercially known as Oberon^®^, was developed for the control of whiteflies and spider mites and has become a key component in resistance management programs for crops [31,32]. Environmentally, spiromesifen has a moderate to low impact, with a soil degradation half-life of approximately 5 days, no significant leaching potential, and low water solubility (0.13 mg/mL) [31].

Spiromesifen inhibits the synthesis of triglycerides and free fatty acids by targeting the acetyl-CoA carboxylase, which catalyzes the carboxylation of acetyl-CoA to malonyl-CoA, the first step in fatty acid biosynthesis [33]. Additionally, spiromesifen has been reported to affect carbohydrate and glycogen content [34], which play a crucial role in insect physiology, including flight, molting, and reproduction [35].

Lipid metabolism is essential for maintaining energy balance in mosquitoes and is linked to various physiological processes. *Culex pipiens* and *Aedes albopictus* (Skuse, 1894) use lipids as their main energy reserves during diapause [36,37]. Lipids also provide the necessary energy for oocyte maturation and embryonic development [38]. In *Culex quinquefasciatus* Say, about 90% of the energy utilized by developing embryos is derived from lipids [39]. Similarly, in *Ae. aegypti*, 80% of the lipids found in mature oocytes come from lipids stored in the fat body, synthesized from sugars ingested before a blood meal [40]. Lipid metabolism also plays a crucial role in the infection of mosquitoes with *Plasmodium* and arboviruses [41,42,43,44,45,46,47,48].

Spiromesifen is most effective against the immature stages (larvae and nymphs) of target pests. It interferes with their development, leading to the death of larvae and nymphs before they mature into adults. Insects treated with spiromesifen typically exhibit reduced feeding, impaired growth, and failure to develop into the next life stage [49,50]. In mosquitoes, spiromesifen has demonstrated effects on the immature stages of *Cx. pipiens* and *Culiseta longiareolata* (Macquart, 1838), showing reductions in body volume and the content of carbohydrates, lipids, and proteins. Additionally, exposure to spiromesifen increases malondialdehyde (MDA) levels, a product of lipid peroxidation, as well as catalase (CAT) activity, both biomarkers of oxidative stress [51,52]. In agricultural pests, spiromesifen exposure affects the reproductive parameters of females by reducing fecundity and fertility [53,54,55,56].

This study aimed to explore the biological effects of spiromesifen on larval populations of *Ae. aegypti* resistant to temephos, considering that this lipid inhibitor could be an alternative larvicide in populations resistant to conventional insecticides for larval control. Concentration-response parameters and the effects of exposure to LC_50_ on morphometric measurements and main biochemical components (carbohydrates, lipids, and proteins) were determined. Metabolic responses were assessed by measuring catalase (CAT) activity and malondialdehyde (MDA) levels. Additionally, the sterilizing effect of spiromesifen on female *Ae. aegypti* was evaluated.

## 2. Materials and Methods

### 2.1. Collection and Rearing of Biological Material

Immature stages of *Ae. aegypti* were collected in 2022 from various locations in Nuevo Leon, northeastern Mexico. Populations were collected from the municipalities of Apodaca (25°42′24.5″ N, 100°09′01.8″ W), Guadalupe (25°38′51.3″ N, 100°12′01.6″ W), and Monterrey (25°39′40.0″ N, 100°19′26.5″ W). Between 1300 and 1600 *Ae. aegypti* larvae were collected from at least 10 breeding sites per location. The larvae were then transported to the Medical Entomology Laboratory at the Faculty of Biological Sciences, Universidad Autonoma de Nuevo Leon, Mexico. In the laboratory, larvae were placed in plastic trays with dechlorinated water and fed powdered bovine liver protein (Liver Powder MP Biomedicals, LLC, Santa Ana, CA, USA). Once the larvae reached the pupal stage, they were transferred to 250 mL flasks and kept in cages (30 cm × 30 cm) until the adults emerged. Male mosquitoes were fed a 10% sugar solution, while females were artificially fed with lamb blood (*Ovis orientalis*) to produce eggs. Plastic cups containing dechlorinated water and filter paper as a substrate for oviposition were placed inside adult cages to obtain the F_1_ generation used for bioassays. The biological material was reared under insectary conditions at 28 ± 1 °C and 70 ± 5% relative humidity with a 12:12 h light–dark photoperiod. The New Orleans strain (NO) was used as a susceptible reference in the study, this strain was originally obtained from the CDC (Atlanta, GA, USA) and has been maintained since 2002.

### 2.2. Assessing Temephos Resistance in Ae. aegypti Populations

Bioassays were conducted in late 3rd instar–early 4th-instar larvae to determine the susceptibility to temephos. Larvae were exposed to a discriminant concentration (DC) of 0.012 mg/L of temephos [57] diluted in ethanol (technical grade, 97.5% purity; Chem Service, West Chester, PA, USA) in groups of 25 individuals per replicate (4 replicates) with a control containing 1 mL of ethanol diluted in water (25 individuals). Mortality was recorded after 24 h of exposure. If control mortality ranged from 5% to 20%, Abbott’s formula was applied [58]. In cases where control mortality exceeded 20%, the bioassay was discarded. All the procedures described above were also performed on the susceptible NO strain.

The mortality percentage was calculated at 24 h to determine the presence of resistance. Additionally, the intensity of resistance was analyzed by exposing the populations to five times (5×) the DC (0.06 mg/L) and ten times (10×) the DC (0.12 mg/L) of temephos.

Resistance frequency was calculated using the WHO criteria to categorize the populations as follows: susceptible when mortality was ≥98%; mortality between 90 and 97% suggests possible resistance, requiring confirmation; and mortality < 90% indicates resistance [59]. The results of resistance intensity were interpreted as follows: Mortality ≥ 98% at 5× DC exposure was considered low intensity, and mortality < 98% moderate to high intensity. For 10× DC exposure, mortality ≥ 98% was considered moderate-intensity resistance, and mortality < 98% high-intensity resistance.

### 2.3. Bioassays with Spiromesifen

Bioassays with the insecticide spiromesifen (technical grade, 98% purity; Chem Service, West Chester, PA, USA) were conducted to determine concentration-response parameters in field populations and the susceptible NO strain. Twenty-five newly molted 4th-instar larvae of the F_1_ generation were exposed in four replicates to various concentrations of spiromesifen, ranging from 0.01 to 15 mg/L for the NO strain and from 0.05 to 30 mg/L for the field populations, for 24 h. Controls without insecticide were included for all populations and the NO strain. After exposure, the larvae were rinsed and transferred to cups with water and food (powdered bovine liver protein) to monitor their development. Mortality was recorded daily until adult emergence, and emergence inhibition (EI) was calculated [51,52,59]. If adult emergence in the control was less than 90%, the test was discarded and repeated. However, if the percentage of emergence in the control was between 91% and 99%, it was corrected using Abbott’s formula [58,59].

### 2.4. Effects of Exposure to Spiromesifen

#### 2.4.1. Exposure to LC_50_ of Spiromesifen

Bioassays were conducted by exposing approximately 100 newly molted 4th-instar larvae (F_1_) from each field population and the susceptible NO strain to their respective LC_50_ concentrations of spiromesifen, previously obtained in concentration-response assays. A control group without insecticide was included for each population. After the initial 24 h spiromesifen exposure, the surviving larvae were rinsed and transferred to clean water with food. Groups of surviving larvae were collected at different time points: 24 h, 48 h, and 72 h post-exposure. This process was repeated for the control groups (without insecticide). A total of 30 larvae were collected from each group at each time point. The larvae were individually weighed, measured, and immediately stored at −20 °C for further analysis.

#### 2.4.2. Morphometric Measurements

Morphometric measurements were performed on different groups of larvae exposed to the LC_50_ of spiromesifen, as well as on control groups from the field populations and the susceptible NO strain. Body weight was measured individually using an analytical balance and expressed in milligrams (mg) (Denver Instruments, Bohemia, NY, USA). The thorax width was measured at its widest point, and the measurements were expressed in cubic millimeters (mm^3^) to estimate body volume [60]. The measurements were performed using Image J software (Version 1.53t, National Institutes of Health, Bethesda, MD, USA).

#### 2.4.3. Body Biochemical Composition

Ten individuals per group (24 h, 48 h, and 72 h) were taken from the treatment and control groups of field populations and the susceptible NO strain to quantify carbohydrates, lipids, and proteins. The biochemical analysis of these components was determined using the method adapted by Foray et al. [61], which combines the techniques of van Handel [62,63] and Bradford [64] to simultaneously determine carbohydrates, lipids, and proteins in a single individual. The Bradford [64] technique was employed for protein determination, using bovine serum albumin (Sigma-Aldrich, St. Louis, MO, USA) as the standard. Carbohydrates and lipids were extracted using the procedures described by van Handel and van Handel & Day [65,66]. Total carbohydrate content was determined by the anthrone method as described by van Handel [62,65], using glucose as the standard. Total lipid content was determined and measured using the vanillin assay, with glyceryl trioleate (Sigma-Aldrich, St. Louis, MO, USA) as the standard [63]. The total content of carbohydrates, lipids, and proteins was determined individually per larva and expressed as a function of larval body weight (μg/mg of larvae) to allow for precise comparisons between the control and treated groups, considering the reduction in larval weight and volume. For specific methodological details and complete experimental procedures, refer to Foray et al. [61].

#### 2.4.4. Determination of Catalase (CAT) Activity and Malondialdehyde (MDA)

Catalase (CAT) activity was determined in 10 treated larvae per group (24 h, 48 h, and 72 h) and their controls across all populations and the NO strain using the modified spectrophotometric method [67]. The larvae were homogenized in phosphate buffer (0.1 M, pH 7.4), and the enzymatic reaction was initiated by adding hydrogen peroxide (H₂O₂) to the homogenate. The decrease in absorbance at 240 nm, corresponding to the decomposition of H₂O₂, was monitored. Enzymatic activity was expressed in μmol/min/mg of protein.

To measure MDA levels, ten individuals from each group, both treatment and control (24 h, 48 h, and 72 h), were selected from all populations. The thiobarbituric acid reactive substances (TBARS) assay was used to measure MDA levels [68]. In this assay, MDA reacts with thiobarbituric acid under acidic conditions and high temperatures to form a pink MDA-TBA complex, the intensity of which is measured spectrophotometrically at 532 nm. Absorbances were measured using a spectrophotometer (ASYS Hitech GmbH, Eugendorf, Austria), and the values obtained were expressed in micromoles of MDA per milligram of protein (µmol/mg of protein).

### 2.5. Evaluation of Sterilizing Properties

The impact of spiromesifen on the fecundity of *Ae. aegypti* was evaluated using the WHO protocol for assessing the sterilizing properties of pyriproxyfen [69]. One hundred 5-day-old blood-fed females from each field population (Apodaca, Guadalupe, Monterrey) and the susceptible NO strain were exposed to spiromesifen in groups of 25. These females were kept in cages with sufficient males from the moment of their emergence. Wheaton bottles were coated with the LC_50_ and LC_99_ of spiromesifen (active ingredient diluted in acetone) obtained in larval bioassays for each field population and the susceptible NO strain. Control groups were exposed to acetone only. The females were exposed for 1 h and then transferred to paper cups covered with mesh, with 10% sucrose-soaked cotton provided. The cups were maintained at 28 ± 1 °C and 70 ± 5% RH with a 12:12 h L:D photoperiod for 72 h, during which mortality was recorded every 24 h.

After 72 h, each surviving female was individually transferred to new paper cups containing 30 mL of water. The females were provided with 10% sucrose solution cotton, which was replaced daily. The cups were kept under the previously described conditions for four days, after which the number of eggs laid by the treatment and control groups (unexposed) of the NO strain and field populations was recorded. The test exclusion criteria were (a) mortality in the control groups >20% at 72 h post-exposure, (b) oviposition rate in the control groups ≤30% at the end of day seven, and (c) oviposition inhibition in the susceptible strain at the end of day seven after 1 h of insecticide exposure < 98% [69].

The oviposition rate was determined by the proportion of females that laid eggs among those that survived spiromesifen exposure. Oviposition inhibition was determined by comparing the proportion of females that laid eggs in the treatment group to the control group. Fecundity was measured by the average number of eggs laid per female, and the inhibition (%) in fecundity was calculated as the reduction in the number of eggs laid per female in the treatments (LC_50_ and LC_99_) compared to the control. Fertility was determined by the number of larvae hatched per number of eggs laid, and fertility inhibition by the proportion of eggs hatched in the treatment compared to the control group.

Additionally, the total carbohydrate and lipid content in females exposed to LC_50_ and LC_99_ of spiromesifen was quantified using the methodology previously described in larval bioassays. Each parameter was analyzed in groups exposed to LC_50_ and LC99 and the control groups.

### 2.6. Statistical Analysis

The results of the concentration-response bioassays with spiromesifen were subjected to log-probit regression analysis (PoloPlus 2.0, LeOra Software, Berkeley, CA, USA). Lethal concentrations 50 and 90 (LC_50_ and LC_90_) were determined for each population and the NO strain. Additionally, the LC_99_ was calculated and subsequently used for the bioassays with adult females. LC values with non-overlapping confidence intervals were significantly different. Resistance to spiromesifen was determined by the resistance ratio (RR), calculated by dividing the LC_50_ value of the field populations by the LC_50_ value of the NO strain. The Mazzari and Georghiou [70] criterion was used to establish resistance: An RR < 5 indicates a susceptible field population, an RR between 5 and 10 indicates moderate resistance, and an RR >10 indicates high resistance. Additionally, resistance was evaluated in relation to the susceptible NO strain at the LC_90_ level.

The results for body weight; body volume; carbohydrate, lipid, and protein content; and oxidative stress biomarkers (CAT and MDA) are expressed as mean ± SEM for the treated larval and control groups of the field populations and the susceptible NO strain. The significance of differences between the treated groups and their controls at each time (24 h, 48 h, 72 h) was tested using the Mann–Whitney U test. Female mortality, oviposition rates, fecundity, fertility, and lipid and carbohydrate content were compared within each strain/population between the control and exposure to LC_50_ and LC_99_ using the Kruskal–Wallis test, followed by Dunn’s multiple comparisons tests. The significance level for all analyses was set at α = 0.05. All analyses were performed using GraphPad Prism v.9 (GraphPad Software, Inc., Version 9.0, La Jolla, CA, USA).

## 3. Results

### 3.1. Susceptibility to Temephos

Our analysis revealed that the three field populations of *Ae. aegypti* exhibited resistance to temephos, with mortality rates ranging from 10% to 68% when exposed to the diagnostic concentration (DC) of 0.012 mg/L. These field populations showed a moderate intensity of resistance, with mortality rates of 92% to 94% when exposed at 5× DC and 100% at 10× DC (Table 1).

### 3.2. Susceptibility to Spiromesifen

The concentration-response relationship for spiromesifen on newly molted fourth-instar larvae was determined for both field populations and the susceptible NO strain. Mortality (emergence inhibition) was recorded until adult emergence. The LC_50_ value for the Monterrey population (4.02 mg/L) was significantly higher compared to the values for the Guadalupe population (1.81 mg/L); however, it did not differ from the LC_50_ value of the Apodaca population (3.41 mg/L) and the susceptible NO strain (1.12 mg/L). Regarding the LC_90_ values, no population differed significantly from the NO strain (48.60 mg/L) (*p* < 0.05). The resistance ratios indicated that the Guadalupe, Apodaca, and Monterrey populations were susceptible to spiromesifen, with RR_50_ and RR_90_ values of less than 5 (Table 2).

### 3.3. Effects of LC_50_ of Spiromesifen in Larvae

#### 3.3.1. Effects of Exposure to LC_50_ of Spiromesifen on Body Weight and Volume

**Figure 1:**
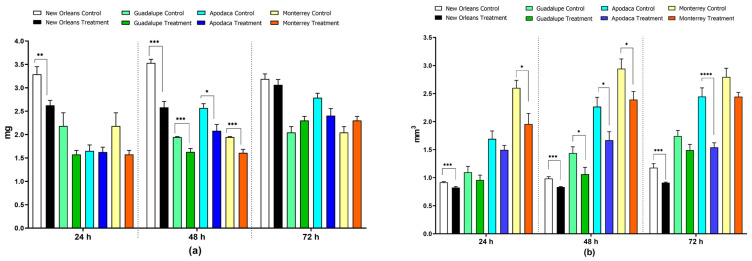
Effect of LC_50_ spiromesifen exposure on (**a**) body weight (mg) and (**b**) body volume (mm^3^) on 4th instar *Ae. aegypti* larvae over time (mean ± SEM; * *p* < 0.05, ** *p* < 0.01, *** *p* < 0.001, **** *p* < 0.0001; Mann–Whitney U Test).

*Ae. aegypti* larvae exhibited differential effects on body weight over three time intervals following exposure to the LC_50_ of spiromesifen. Twenty-four hours post-exposure, a significant decrease in larval body weight was observed only in the susceptible NO strain, with a 20% reduction from 3.29 ± 0.17 mg in the control group (C) to 2.63 ± 0.11 mg in the treated group (T) (*p* < 0.01). At 48 h, a significant reduction in body weight was noted across all populations. The greatest reduction was seen in the susceptible NO strain, with a 27% decrease from 3.53 ± 0.08 mg (C) to 2.58 ± 0.13 mg (T) (*p* = 0.0001). This was followed by the Apodaca population, with a 19% reduction (2.57 ± 0.09 mg C to 2.08 ± 0.14 mg T) (*p* < 0.05); the Monterrey population, with a 17% reduction (1.95 ± 0.01 mg C to 1.61 ± 0.08 mg T) (*p* = 0.0001); and the Guadalupe population, with a 16% reduction (1.95 ± 0.0.01 mg C to 1.63 ± 0.07 mg T) (*p* = 0.0001) (Figure 1a; Table S1). These results suggest that spiromesifen effectively reduced larval weight in the susceptible strain in the short term, with a pronounced effect observed at 48 h in field populations.

Exposure to spiromesifen also caused a significant reduction in body volume. At 24 h, the Monterrey population exhibited a 25% reduction in body volume (2.6 ± 0.13 mm^3^ C to 1.96 ± 0.19 mm^3^ T) (*p* < 0.05), and the susceptible NO strain showed a 10% reduction (0.92 ± 0.01 mm^3^ C to 0.83 ± 0.02 mm^3^ T) (*p* < 0.0001). At 48 h, significant reductions were observed in all populations: a 15% reduction in the NO strain (0.99 ± 0.03 mm^3^ C to 0.83 ± 0.01 mm^3^ T) (*p* < 0.0001), a 26% reduction in the Apodaca population (2.27 ± 0.17 mm^3^ C to 1.67 ± 0.15 mm^3^ T) (*p* < 0.05), a 26% reduction in the Guadalupe population (1.44 ± 0.11 mm^3^ C to 1.06 ± 0.12 mm^3^ T) (*p* < 0.05), and a 19% reduction in the Monterrey population (2.95 ± 0.17 mm^3^ C to 2.39 ± 0.14 mm^3^ T) (*p* < 0.05). At 72 h, the effect persisted in the susceptible strain, with a 23% reduction in body volume (1.18 ± 0.07 mm^3^ C to 0.91 ± 0.01 mm^3^ T) (*p* < 0.0001), and in the Apodaca population, with a 37% reduction (2.45 ± 0.15 mm^3^ C to 1.54 ± 0.08 mm^3^ T) (*p* < 0.0001) (Figure 1b; Table S2).

#### 3.3.2. Effect of Spiromesifen on the Biochemical Composition of Larvae

The effects of spiromesifen on the biochemical composition of newly molted fourth-instar larvae of *Ae. aegypti* were evaluated by examining the carbohydrate, lipid, and protein content of the larvae exposed to the LC_50_ in both temephos-resistant populations and the susceptible NO strain.

Regarding total carbohydrate content, a significant reduction was observed in the NO strain and the Apodaca and Monterrey populations at 24 h compared to the control groups. The greatest reduction in carbohydrate content was observed in the Apodaca population, with a 56% decrease compared to its control (*p* < 0.0001), followed by the Monterrey population, with a 40% reduction, and the Guadalupe population and the susceptible NO strain, with 27% reductions (*p* < 0.01). At 48 h, this effect was only evident in the susceptible strain, showing a 29% reduction compared to the control (*p* < 0.01). At 72 h, the effect was evident in both the susceptible NO strain and the Monterrey population, with carbohydrate content reductions of 46% and 13%, respectively, compared to their respective controls (*p* < 0.05) (Table 3 and Table S3).

For lipids, a significant reduction in content was observed in the larvae from the Apodaca population at 24 h (*p* < 0.05). At 48 h, this effect was observed in all populations and the susceptible NO strain compared to their controls. The greatest reduction in lipid content was observed in the Apodaca population, with a 65% decrease (*p* < 0.0001), followed by Monterrey, with a 34% reduction (*p* < 0.05); the susceptible NO strain, with a 26% reduction (*p* < 0.001); and the Guadalupe population, with a 23% reduction (*p* < 0.05). The significant reduction continued at 72 h, with reductions of 39% for the NO strain (*p* < 0.05), 29% for the Apodaca population (*p* < 0.001), and 24% for the Monterrey population (*p* < 0.005) (Table 3 and Table S4).

The reduction in total protein content became evident at 72 h for all populations, except for the NO strain, compared to their respective controls. The magnitude of reduction was the same for the Guadalupe and Apodaca populations, at 23% (*p* < 0.01), while the Monterrey population showed a smaller reduction of 15% (*p* < 0.05) (Table 3 and Table S5).

#### 3.3.3. Effects of Spiromesifen on Oxidative Stress Biomarkers in *Ae. aegypti* Larvae

**Figure 2:**
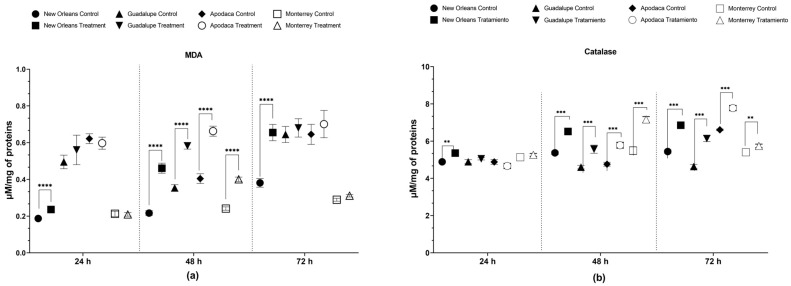
Effects of LC_50_ spiromesifen exposure on oxidative stress biomarkers. (**a**) Malondialdehyde (MDA) and (**b**) catalase (CAT) in fourth-instar *Ae. aegypti* larvae over time (mean ± SEM; ** *p* < 0.01, *** *p* < 0.001, **** *p* < 0.0001; Mann–Whitney U Test).

Exposure to spiromesifen significantly increased MDA levels at 48 h in all populations and the susceptible NO strain compared to the control groups. The NO strain showed a 113% increase in MDA levels, while the other populations exhibited increases of between 64% and 66%. This effect was recorded only at 24 h for the NO strain and persisted at 72 h exclusively for this strain (*p* < 0.0001) (Figure 2a; Table S6).

Spiromesifen also induced a significant increase in CAT activity, starting at 24 h, with a 9.6% increase observed only in the susceptible NO strain (*p* < 0.05). This effect was generalized across the susceptible strain and all field populations at 48 h, with increases ranging from 21% to 30% (*p* < 0.001). The increase in CAT activity persisted at 72 h in all populations, with a 32% increase in the Guadalupe population, a 26% increase in the susceptible strain, and 18% and 6% increases in the Apodaca and Monterrey populations, respectively, compared to their controls (*p* < 0.01) (Figure 2b; Table S7). These results confirm that spiromesifen exerted the greatest effect on oxidative stress biomarkers starting at 48 h.

### 3.4. Effects of Spiromesifen in Ae. aegypti Adult Females

#### 3.4.1. Sterilizing Effect of Spiromesifen

The effects of spiromesifen on the fecundity and fertility of *Ae. aegypti* females were evaluated using the WHO protocol to assess the sterilizing properties of pyriproxyfen [69]. The oviposition rate and percentage of oviposition inhibition were determined by exposing females to the LC_50_ and LC_99_ concentrations obtained in previous larval bioassays. Detailed results for mortality, oviposition, fecundity, and fertility are presented in Table 4 and Tables S8 and S9.

Exposure of *Ae. aegypti* females to LC_99_ resulted in significantly higher mortality levels than the control for the susceptible NO strain and the field populations (*p* < 0.05). No significant difference in mortality was found when females were exposed to the LC_50_ (*p* > 0.05). The highest inhibition of oviposition was 66% in females from the Monterrey population exposed to LC_99_, followed by 65% in the susceptible NO strain, 53% in the Guadalupe population, and 51% in the Apodaca population. Exposure of females to LC_50_ caused a reduction in oviposition of 36% for the susceptible NO strain and the Apodaca and Monterrey populations and only 22% in the Guadalupe population (Table 4).

**Figure 3:**
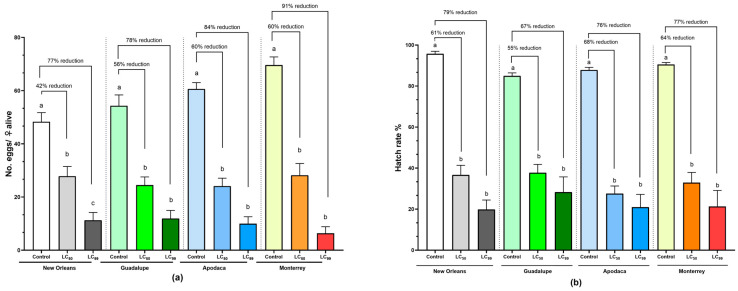
Fecundity and egg fertility (mean ± SEM) of *Ae. aegypti* females exposed to LC_50_ and LC_99_ of spiromesifen. (**a**) Number of eggs per female alive. (**b**) Percentage of egg hatchability. Different letters in each bar indicate significant differences within each strain/population (mean ± SEM; Kruskal–Wallis test followed by Dunn’s multiple comparisons test, *p* < 0.05).

When evaluating fecundity inhibition, calculated from females that survived spiromesifen exposure, significant effects were observed at both LC_50_ and LC_99_ compared to the control. Exposure to LC_50_ resulted in a fecundity inhibition of 42% in the susceptible NO strain (*p* < 0.01), 56% in the Guadalupe population, and 60% in the Apodaca and Monterrey populations (*p* < 0.0001). On the other hand, exposure to LC_99_ induced greater fecundity inhibition than LC_50_, reaching 77% in the susceptible NO strain, and 78%, 84%, and 91% in the Guadalupe, Apodaca, and Monterrey populations, respectively (*p* < 0.0001) (Table 4, Figure 3a). When reported as the number of females contributing to egg-laying in each group, a significant reduction in female fecundity was observed at LC_50_ and LC_99_ compared to their respective controls for all field populations (*p* < 0.0001). However, for the susceptible NO strain, no difference was found (*p* > 0.05) (Table 4).

The average hatching rate differed significantly in groups exposed to LC_50_ and LC_99_ compared to the control for all populations and the susceptible strain (*p* < 0.0001). No significant difference in hatching rate was found when comparing the two concentrations across all populations (*p* > 0.05). Fertility inhibition ranged from 55% to 68% when exposed to LC_50_ and from 67% to 79% when exposed to LC_99_ (Table 4, Figure 3b).

#### 3.4.2. Carbohydrate and Lipid Contents in Females Exposed to LC_50_ and LC_99_ of Spiromesifen

The effects of spiromesifen on the carbohydrate and lipid contents in *Ae. aegypti* females exposed to LC_50_ and LC_99_ of spiromesifen were also evaluated. The total carbohydrate content was significantly reduced when females from the Apodaca and Monterrey populations were exposed to LC_50_ of spiromesifen (*p* < 0.05). However, when females were exposed to LC_99_, the reduction in carbohydrate content was significant for the Apodaca and Monterrey populations and the susceptible NO strain (*p* < 0.001), as well as the Guadalupe population (*p* < 0.05). In contrast to carbohydrates, the total lipid content was significantly reduced for all populations (*p* < 0.01) and the susceptible NO strain (*p* < 0.05) when exposed to LC_50_. This reduction was also significant for all populations and the susceptible strain when females were exposed to LC_99_ (*p* < 0.001) (Table 5 and Tables S10 and S11).

## 4. Discussion

The increasing insecticide resistance in *Ae. aegypti* presents a major challenge for vector control, leading us to evaluate the efficacy of spiromesifen, a lipid synthesis inhibitor.

A key part of our study was the initial assessment of temephos resistance in local *Ae. aegypti* populations, which laid the groundwork for selecting populations for subsequent spiromesifen experiments. Temephos has been used since 1969 in Canada, Ecuador, the United States, and Mexico [71]. However, resistance to this insecticide has been found in *Ae. aegypti* populations across several Latin American and Caribbean countries [72]. In Mexico, Dávila-Barboza et al. [10] recently analyzed 23 populations of *Ae. aegypti* from different regions, showing that 78% of these populations exhibited moderate resistance to temephos, and 39% showed high resistance intensity. This confirms a high prevalence of temephos resistance nationwide and underlines the urgent need to review current larval control strategies.

Our results align with these findings, as the *Ae. aegypti* populations included in our research showed high frequencies of temephos resistance, with mortality rates of between 10% and 68% after exposure to the DC of 0.0125 mg/L, and moderate resistance intensity with less than 98% mortality when exposed to 5× DC. Although temephos is not the only larvicide recommended for mosquito control in Mexico, it represents a low-cost control agent that has been used in dengue control campaigns for more than 50 years [10].

Considering that spiromesifen is an alternative insecticide in resistance management programs for agricultural pests [32], our study evaluated the larval susceptibility of temephos-resistant *Ae. aegypti* to this insecticide. The three populations of *Ae. aegypti* showed susceptibility to spiromesifen, with RR_50_ and RR_90_ values lower than four-fold. Few studies have analyzed spiromesifen susceptibility in mosquitoes; however, a study on *Culex quinquefasciatus* reported an LC_50_ value of 0.542 mg/L and an LC_90_ value of 1.148 mg/L for the active ingredient in newly molted fourth-instar larvae using a commercial formulation, Oberon^®^ 240 SC [51]. This is important to consider since formulated products often contain adjuvants and other ingredients that enhance the effectiveness of the insecticide [73]. Similar studies in *Cs. longiareolata* showed an LC_50_ value of 0.555 mg/L and an LC_90_ value of 1.366 mg/L for spiromesifen [52].

Spiromesifen (a.i., 98% purity) was used in our study, yielding LC_50_ values of between 1.12 and 4.02 mg/L and LC_90_ values of between 17.59 and 63.65 mg/L for the susceptible NO strain and the three field populations of *Ae. aegypti*. Marina et al. [74] reported LC_50_ values of 6 mg a.i./L for spiromesifen for *Ae. aegypti* larvae using the commercial formulation Oberon 240 SC, which is comparatively higher than the values obtained for our populations, as the highest LC_50_ value recorded for the Monterrey population was 4.02 mg/L. However, these results should be taken with caution, as the authors recorded mortality 48 h after the start of the bioassay, following a 24 h period of exposure to the insecticide. Since this insecticide is a lipid synthesis inhibitor and affects growth and development in juvenile stages, larval mortality or disruption in development extends beyond 48 h [49,50]. This aligns with observations from the same authors, who indicated that mortality did not reach 90% in the bioassay with spiromesifen at 24 or 48 h post-treatment.

Exposing *Ae. aegypti* larvae to LC_50_ values of 1.12 mg/L, 1.18 mg/L, 3.41 mg/L, and 4.02 mg/L for the susceptible NO strain and the field populations of Guadalupe, Apodaca, and Monterrey, respectively, significantly reduced the total carbohydrate, lipid, and protein contents. Initially, the total content of these biochemical components was measured individually per larva. However, given the observed reduction in larval weight and volume in the treated groups, we expressed these biochemical contents as a function of larval body weight (µg/mg of larvae) to ensure a more precise comparison. This normalization accounts for the variability in larval weight, which can significantly impact the absolute amounts of these biochemical components. By expressing the contents relative to larval weight, we aimed to provide a more accurate representation of the biochemical changes induced by the treatment, independent of size-related variations.

A reduction in total carbohydrate content was observed in larvae from the NO strain and field populations of *Ae. aegypti*, with significant decreases ranging from 27% to 56% at 24 h of treatment. This initial reduction could be attributed to carbohydrates being the first energy reserves used by the insect to recover from the stress induced by insecticide exposure. Similar findings have been reported in *Cx. quinquefasciatus* larvae exposed to an LC_90_ of spiromesifen at 1.148 mg/L, where a significant 29% reduction in carbohydrate content was observed, while no effect was noted at the LC_50_ of 0.542 mg/L [51]. Further supporting these observations, it has been demonstrated that the topical application of spiromesifen on *Drosophila melanogaster* pupae significantly decreased carbohydrate and glycogen levels [34].

Carbohydrates play a key role in the energy metabolism and overall physiology of insects. As the primary source of readily available energy, they are essential for various metabolic and physiological activities. In situations of high energy demand, such as flight, migration, and stress, carbohydrates stored as glycogen in the insect body are quickly mobilized to meet these demands [75]. Furthermore, carbohydrates are vital for embryonic development and larval growth, providing the necessary energy during these critical periods [39]. During starvation or environmental stress, insects rely on their glycogen reserves to maintain vital functions and survive [36]. Thus, the significant reduction in carbohydrate content following spiromesifen exposure reaffirms the findings of other authors [34,51,52] and highlights its impact on the energy metabolism of *Ae. aegypti* larvae.

When evaluating the effect on lipid content after exposure to the LC_50_ of spiromesifen, we found a significant reduction in lipid levels in all evaluated populations at 48 h of treatment, ranging from 23% to 65%. This time point was identified as optimal for exerting the greatest impact on total lipid content. The reduction in lipid content could be attributed not only to the inhibition of lipogenesis by the insecticide but also to the insect’s metabolic response, where carbohydrates are used as the primary energy source [75]. Once these reserves are depleted, the insect turns to lipid reserves for energy [76,77], which explains the significant reduction in lipid content observed at 48 h. This effect continued at 72 h for the susceptible strain and the Apodaca and Monterrey populations.

Studies conducted on *Cs. longiareolata* have demonstrated that spiromesifen, at an LC_50_ of 0.555 mg/L and an LC_90_ of 1.366 mg/L, reduces total lipid content and increases MDA levels, a biomarker indicating oxidative damage to lipid molecules [52]. The formation of MDA as a result of lipid peroxidation serves as a marker of oxidative stress due to its ability to indicate oxidative damage in cells [78]. Our results are consistent with these findings, as an increase in MDA levels was recorded from 48 h onwards in both the susceptible strain and field populations. Additionally, from 48 h and continuing to 72 h, catalase activity significantly increased in larvae treated with the LC_50_ of spiromesifen in all populations. Increases in catalase levels indicate an insect defense mechanism to counteract oxidative stress in response to insecticide exposure [79].

An increase in lactate dehydrogenase (LDH) activity has also been observed in *D. melanogaster* pupae exposed to spiromesifen. LDH is a key enzyme in glycolysis, and its increase indicates that the insect is converting pyruvate to lactate to meet the high energy demand caused by chemical stress [34].

Lipids are the main fat component of the body, with more than 90% of stored lipids being triglycerides synthesized from dietary carbohydrates, fatty acids, or proteins [80,81]. They are one of the most important reserves and are involved in many essential functions of the insect. During the larval stage, lipids are stored in the fat body and mobilized to meet energy needs during growth and metamorphosis. Lipid reserves are crucial for larva survival and successful development, providing the necessary energy for intensive metabolic processes and the transition to the adult stage [40]. Additionally, the amount of nutrients stored in the larvae has significant consequences for adult life, as a smaller size results in reduced fecundity [82].

Another biochemical component evaluated after exposure to the LC_50_ of spiromesifen was total protein content, which showed a significant reduction in the field populations at 72 h. This delayed reduction compared to carbohydrates at 24 h and lipids at 48 h can be explained by the different rates of utilization and depletion of these components. Initially, larvae use their carbohydrate reserves, a readily available energy source; once these reserves are exhausted, they turn to lipids for energy. Finally, proteins, which play an essential role in cellular structure synthesis and vital metabolic functions, are used as the last energy reserve, leading to a significant reduction in protein content at 72 h [83,84]. Our results align with studies on *Cx. pipiens* and *Cs. longiareolata*, which reported reductions in total protein content only at higher concentrations (LC_90_: 1148.65 µg/L and 1366.70 µg/L, respectively) [51,52].

The reduction in essential biochemical components—carbohydrates, lipids, and proteins—negatively impacted the growth and development of the larvae. Energy reserves are essential for growth, and the decrease in these reserves due to exposure to the LC_50_ of spiromesifen resulted in a significant reduction in larval body weight and volume for all populations at 48 h, as well as in the susceptible NO strain at all three evaluation times. Similar findings have been reported in *D. melanogaster* pupae treated with a dose of 35.53 µg/insect of spiromesifen via topical application, where a significant reduction in weight was observed [34]. Body weight and volume reductions in *Cx. quinquefasciatus* larvae have also been reported after exposure to LC_50_ of 0.542 mg/L and LC_90_ of 1.366 mg/L of spiromesifen [51].

The importance of nutrient storage to the survival and development of *Ae. aegypti* is well recognized. Nutrient storage is essential for various aspects of their physiology, including maintaining metabolic activity and providing energy for prolonged flight, oogenesis, and starvation resistance [75,85,86,87]. Additionally, intracellular lipid trafficking plays a significant role in mosquitoes and dengue virus (DENV) infection dynamics [88,89,90]. Stored nutrients are not only vital for growth and development but also for pathogen replication and the cell’s ability to generate an immune response [91].

An additional objective of this study was to investigate the impact of spiromesifen exposure on the fecundity and fertility of *Ae. aegypti* females. This assessment was motivated by the potential use of spiromesifen as a larvicide for this mosquito species. We hypothesized that females might be indirectly exposed by laying eggs in breeding sites treated with spiromesifen, possibly leading to a sterilizing effect similar to that of pyriproxyfen. To evaluate this, we followed the WHO protocol [69] for assessing the sterilizing properties of pyriproxyfen and applied the LC_50_ and LC_99_ of spiromesifen obtained from larval bioassays. Our findings revealed that spiromesifen inhibited oviposition, with reductions of 22% to 36% at LC_50_ and 51% to 66% at LC_99_. Furthermore, the insecticide led to a notable decrease in fecundity, with inhibition ranging from 42% to 60% at LC_50_ and from 77% to 91% at LC_99_. Fertility was also adversely affected, showing reductions of 55% to 68% at LC_50_ and of 67% to 79% at LC_99_. Although no previous studies have specifically investigated the sterilizing effects of spiromesifen on *Ae. aegypti* or mosquitoes, our results suggest a stronger impact on fecundity and fertility compared to pyriproxyfen. Yadav et al. [92] reported 32% fecundity inhibition and 54.3% fertility inhibition when blood-fed females were exposed to a concentration of 0.75% pyriproxyfen. In contrast, spiromesifen concentrations used in our study were much lower, ranging from 0.000112% to 0.000402% for LC_50_ and from 0.011247% to 0.10483% for LC_99_.

The effect of spiromesifen on reducing oviposition rates and egg fertility has been documented in *Bemisia tabaci* (Gennadius, 1889) (Hemiptera: Aleyrodidae), *Tetranychus urticae* Koch (Acari: Tetranychidae), and *Bactericera cockerelli* (Šulc, 1909) (Hemiptera: Triozidae) [53,54,55]. In *D. melanogaster*, exposure to sublethal doses of spiromesifen inhibited ovarian growth and development, reducing the number of oocytes, the volume of basal oocytes, and ovarian weight. Spiromesifen also reduced ovarian levels of carbohydrates and glycogen [56].

Additionally, we investigated whether spiromesifen could affect the carbohydrate and lipid contents of females exposed to LC_50_ and LC_99_. The results showed significant carbohydrate reductions, ranging from 21% to 41% at LC_50_ and from 29% to 54% at LC_99_. For lipids, the reduction was from 21% to 42% at LC_50_ and from 29% to 52% at LC_99_. This is relevant considering that the novo lipogenesis is active in females, as is the accumulation of glycogen after feeding on sugar sources and blood [40,93,94]. Furthermore, the mobilization of lipids to developing oocytes has been demonstrated [95], constituting up to 30–40% of the dry weight of the oocyte [39,82,96]. These findings are significant because any alteration in the females’ ability to synthesize and transfer lipids could directly impact their fecundity and fertility. Therefore, evaluating the impact of spiromesifen not only on larval mortality but also on metabolic processes in adult females could provide a more comprehensive understanding of its effectiveness and mechanism of action as a control agent.

A limitation of our study is its exclusive focus on laboratory conditions, which, while allowing for controlled analysis of spiromesifen’s biological effects, does not provide data on its field efficacy. Nevertheless, our findings demonstrate that spiromesifen significantly impacts not only larval development and biochemical profiles but also the reproductive potential of adult *Ae. aegypti* females. Exposure to spiromesifen resulted in substantial reductions in fecundity and fertility, with inhibition levels reaching up to 91% at the LC_99_ concentration. Additionally, we observed marked decreases in the carbohydrate and lipid contents of females exposed to spiromesifen, which are essential for energy metabolism and oocyte development. These results suggest that spiromesifen disrupts critical metabolic processes, making it a promising candidate for mosquito control across multiple life stages. However, before its implementation in the field, several challenges must be addressed, including optimizing the formulation, dosage, and application frequency to ensure efficacy while minimizing environmental impacts. It is also important to consider additional factors that may influence spiromesifen’s suitability for mosquito control in aquatic habitats. These include its persistence in water, breakdown products, and sensitivity to environmental factors such as sunlight and ambient pH, which could affect its efficacy and environmental impact. Additionally, the toxicity of spiromesifen to non-target aquatic invertebrates and vertebrates is a critical consideration for its use in mosquito control programs. These aspects warrant further investigation to fully understand the potential benefits and risks associated with spiromesifen in field applications. Furthermore, the long-term ecological and evolutionary implications of using spiromesifen need careful consideration, particularly the potential for resistance development and effects on non-target organisms within aquatic ecosystems. Future field trials are necessary to validate these laboratory results and to determine the practical application of spiromesifen within integrated vector management strategies, especially in areas with high levels of insecticide resistance.

## 5. Conclusions

The results of this study reveal a promising breakthrough in the battle against *Ae. aegypti* resistance. The temephos-resistant larval populations exhibited significant susceptibility to spiromesifen, which not only disrupted carbohydrate, lipid, and protein levels but also led to notable reductions in larval weight and volume. Beyond larval impact, spiromesifen dramatically reduced fecundity and fertility in adult females, decreasing lipid and carbohydrate reserves. These findings highlight the remarkable potential of spiromesifen to improve vector control strategies. By leveraging alternative modes of action, such as those employed by spiromesifen, we can target resistant *Ae. aegypti* populations more effectively. Integrating spiromesifen into existing control strategies could be the game-changer needed to overcome insecticide resistance and significantly improve the efficacy of control interventions.

## Figures and Tables

**Table 1:** Frequency and intensity of resistance to the diagnostic concentration (DC) of temephos (0.012 mg/L) and at five times (5×) the DC (0.6 mg/L) and ten times (10×) the DC (0.12 mg/L) in larval populations of *Ae. aegypti* from Nuevo Leon, northeastern Mexico.

Strain/Population	N	Mortality (%)			Status	Intensity of Resistance
		DC	5× DC	10× DC		
New Orleans	300	100	100	100	Susceptible	Susceptible
Apodaca	300	10	94	100	Resistant	Moderate
Guadalupe	300	68	94	100	Resistant	Moderate
Monterrey	300	36	92	100	Resistant	Moderate

N: number of larvae bioassayed.

**Table 2:** Lethal concentrations (LC_50_ and LC_90_) in mg/L and resistance ratio (RR) values in *Ae. aegypti* larvae exposed to spiromesifen.

Strain/ Population	N ^1^	LC_50_ (IC) ^2^	LC_90_ (IC) ^2^	LC_99_ (CI) ^2^	Slope ± SE	X^2^ (df)	*p* Value	RR_50_^3^	RR_90_^3^
New Orleans	1100	1.12 (0.42–2.75) ab	48.60 (14.43–509.66) a	1048.30 (151.36–61412.00) a	0.783 (0.043)	82.13 (9)	0.00	-	-
Guadalupe	1300	1.81 (1.211–2.54) a	17.59 (11.31–32.92) a	112.47 (54.43–342.10) a	1.297 (0.064)	47.39 (11)	0.00	1.49	0.36
Apodaca	1300	3.41 (1.89–5.62) ab	63.65 (29.92–239.45) a	691.69 (195.21–7404.40) a	1.008 (0.057)	71.01 (11)	0.00	2.82	1.30
Monterrey	1500	4.02 (2.69–5.89) b	39.53 (22.67–93.46) a	254.96 (104.98–1092.69) a	1.291 (0.061)	83.56 (13)	0.00	3.31	0.81

^1^ Number of larvae assayed. ^2^ LC_50,_ LC_90_, and LC_99_ represent the concentrations (mg/L) required to kill 50%, 90%, and 99% of 4th-instar larvae, respectively; 95% confidence intervals (CI) are shown in parentheses. ^3^ Resistance ratios were calculated as the LC_50_ or LC_90_ field strain/LC_50_, LC_90_ of the New Orleans strain. Different letters in the columns indicate significant differences.

**Table 3:** Effects of LC_50_ spiromesifen exposure on the content of carbohydrates, lipids, and proteins (μg/mg of larvae) in fourth-instar *Ae. aegypti* larvae over time (mean ± SEM).

Biochemical Content	New Orleans Control	New Orleans LC_50_	Guadalupe Control	Guadalupe LC_50_	Apodaca Control	Apodaca LC_50_	Monterrey Control	Monterrey LC_50_
Carbohydrate								
24 h ^1^	27.23 ± 2.08	19.75 ± 1.22 ^2^**	34.66 ± 5.04	22.80 ± 2.26	48.37 ± 3.90	21.16 ± 3.17 ****	23.20 ± 1.47	13.85 ± 1.74 **
48 h	23.80 ± 1.18	16.93 ± 1.39 **	43.59 ± 2.13	45.51 ± 5.18	30.61 ± 2.13	30.58 ± 2.28	11.28 ± 0.81	12.29 ± 0.98
72 h	28.54 ± 3.79	15.40 ± 1.11 **	61.24 ± 8.11	50.09 ± 6.98	27.08 ± 2.07	21.63 ± 1.60	15.11 ± 0.70	13.13 ± 0.55 *
Lipid								
24 h	29.11 ± 3.13	25.10 ± 2.40	44.01 ± 5.03	46.26 ± 3.36	47.39 ± 4.96	32.10 ± 3.42 *	22.43 ± 1.36	24.26 ± 3.29
48 h	29.01 ± 2.12	21.50 ± 0.93 ***	71.46 ± 3.91	54.69 ± 4.73 *	39.89 ± 2.57	14.10 ± 1.34 ****	9.51 ± 0.87	6.26 ± 0.67 *
72 h	24.43 ± 3.16	14.90 ± 0.86 *	53.58 ± 6.81	44.77 ± 5.08	15.84 ± 0.72	11.19 ± 0.78 ***	12.80 ± 0.64	9.68 ± 0.56 **
Protein								
24 h	69.20 ± 6.37	78.36 ± 5.23	137.10 ± 16.49	168.10 ± 13.00	162.10 ± 13.01	143.60 ± 15.62	85.99 ± 4.16	106.10 ± 14.00
48 h	58.10 ± 2.70	73.36 ± 2.81 **	127.10 ± 3.09	147.00 ± 9.28	102.50 ± 5.40	99.08 ± 5.40	46.82 ± 3.48	52.01 ± 4.48
72 h	58.28 ± 5.18	44.73 ± 1.77	136.90 ± 12.35	104.80 ± 3.12 **	66.09 ± 2.32	50.79 ± 1.66 ****	61.71 ± 2.88	52.19 ± 2.31 *

^1^ Time of evaluation of the biochemical contents following the exposure to spiromesifen. ^2^ Mann–Whitney U test between the control and treated groups for each strain/population independently at each time after 24 h of exposure: * *p* < 0.05, ** *p* < 0.01, *** *p* < 0.001, **** *p* < 0.0001.

**Table 4:** Effect of spiromesifen on *Ae. aegypti* mortality, oviposition, fecundity, and fertility in a susceptible strain and three temephos-resistant populations.

Strain/Population	New Orleans			Guadalupe			Apodaca			Monterrey		
Treatment	Control	LC_50_	LC_99_	Control	LC_50_	LC_99_	Control	LC_50_	LC_99_	Control	LC_50_	LC_99_
Tested ♀	100	100	100	100	100	100	100	100	100	100	100	100
Mean mortality	4.5 ± 1.2 ^1^ a	11.3 ± 2 a	15.5 ± 1.6 b	4.0 ± 1.3 a	9.0 ± 1.2 a	17.3 ± 2.7 b	3.0 ± 0.7 a	10.5 ± 1.8 a	18.3 ± 0.9 b	3.5 ± 1.3 a	15.8 ± 2.8 a	19.5 ± 1.7 b
% Mortality	18	45	62	16	36	69	12	42	73	14	63	78
Alive	82	55	38	84	64	31	88	58	27	86	37	22
Proportion oviposited (%)	91	58	32	82	64	39	98	62	48	93	59	32
Oviposition inhibition (%)	NA	36	65	NA	22	53	NA	36	51	NA	36	66
Total eggs laid	3959	1530	428	4561	1566	370	5335	1398	269	5986	1042	140
Mean eggs laid (per ♀ that oviposited)	52.8 ± 3.3 a	47.8 ± 3.0 a	35.7 ± 3.5 a	66.1 ± 3.6 a	38.2 ± 3.2 b	30.8 ± 3.4 b	62.0 ± 2.2 a	37.8 ± 2.8 b	22.4 ± 3.4 b	74.8 ± 2.4 a	47.4 ± 3.6 b	20.0 ± 4.6 b
Fecundity (eggs/♀ alive)	48.3 ± 3.4 a	27.8 ± 3.7 b	11.3 ± 2.9 c	54.3 ± 4.1 a	24.5 ± 3.1 b	11.9 ± 3 b	60.6 ± 2.4 a	24.1 ± 3.0 b	10.0 ± 2.6 b	69.6 ± 3.1 a	28.2 ± 4.4 b	6.4 ± 2.5 b
Fecundity inhibition %	NA	42	77	NA	55	78	NA	60	84	NA	60	91
Total eggs hatched	3668	593	83	3814	583	95	4657	475	76	5406	366	42
Mean hatch rate %	95.7 ± 1.2 a	36.7 ± 4.7 b	19.9 ± 4.6 b	85.0 ± 1.5 a	37.7 ± 4.1 b	28.3 ± 7.4 b	87.8 ± 1.3 a	27.5 ± 3.7 b	21.0 ± 6.2 b	90.5 ± 1.0 a	32.9 ± 5.0 b	21.3 ± 7.8 b
Fertility inhibition (%)	NA	61	79	NA	55	67	NA	68	76	NA	64	77

^1^ Mean ± SEM; different letters in each row indicate significant differences among control, LC_50_, and LC_99_ groups within each population or strain according to Kruskal–Wallis analysis (*p* < 0.05).

**Table 5:** Effects of exposure to LC_50_ and LC_99_ of spiromesifen on the content of carbohydrates and lipids (µg) in *Ae. aegypti* females of a susceptible strain and three temephos-resistant populations (mean ± SEM).

Biochemical Content	New Orleans	Guadalupe	Apodaca	Monterrey
Carbohydrate				
Control	61.10 ± 2.26 a ^1^	72.00 ± 2.35 a	75.10 ± 3.26 a	86.90 ± 2.84 a
LC_50_	48.10 ± 2.34 a	54.60 ± 5.06 ab	53.50 ± 5.05 b	50.70 ± 3.98 b
LC_99_	28.20 ± 2.94 b	50.50 ± 6.67 b	40.90 ± 3.85 b	43.50 ± 1.56 b
Lipid				
Control	114.10 ± 5.49 a	94.30 ± 9.97 a	84.20 ± 3.47 a	92.80 ± 5.02 a
LC_50_	81.60 ± 3.39 b	54.10 ± 2.87 b	65.60 ± 2.46 b	55.50 ± 1.80 b
LC_99_	48.60 ± 3.98 b	46.4 ± 4.86 b	59.30 ± 3.18 b	49.00 ± 7.63 b

^1^ Different letters indicate significant differences within each strain/population, Kruskal–Wallis test followed by Dunn’s multiple comparisons test between control and treated groups for each strain/population independently (*p* < 0.05).

## Data Availability

The original contributions presented in the study are included in the article/Supplementary Materials; further inquiries can be directed to the corresponding author.

## Supplementary Materials

The following supporting information can be downloaded at: https://www.mdpi.com/article/10.3390/tropicalmed9080184/s1, Table S1: Weight of *Aedes aegypti* larvae (mg) from temephos-resistant populations and the susceptible New Orleans strain exposed to LC_50_ of spiromesifen.; Table S2: Volume of *Aedes aegypti* larvae (mm^3^) from temephos-resistant populations and the susceptible New Orleans strain exposed to LC_50_ of spiromesifen; Table S3: Carbohydrate content in larvae (µg) of *Aedes aegypti* from temephos-resistant populations and the susceptible New Orleans strain exposed to LC_50_ of spiromesifen; Table S4: Lipid content in larvae (µg) of *Aedes aegypti* from temephos-resistant populations and the susceptible New Orleans strain exposed to LC_50_ of spiromesifen; Table S5: Protein content in larvae (µg) of *Aedes aegypti* from temephos-resistant populations and the susceptible New Orleans strain exposed to LC_50_ of spiromesifen; Table S6: Malondialdehyde (µM/ mg of proteins) in larvae of *Aedes aegypti* from temephos-resistant populations and the susceptible New Orleans strain exposed to LC_50_ of spiromesifen; Table S7: Catalase levels (µM/ mg of proteins) in larvae of *Aedes aegypti* from temephos-resistant populations and the susceptible New Orleans strain exposed to LC_50_ of spiromesifen; Table S8: Number of eggs per female after exposure to LC_50_ and LC_99_ of spiromesifen in adult females of *Aedes aegypti* populations and the susceptible New Orleans strain; Table S9: Number of eggs hatched per eggs oviposited after exposure to LC_50_ and LC_99_ of spiromesifen in adult females of *Aedes aegypti* populations and the susceptible New Orleans strain; Table S10: Carbohydrate content (µg) in females of *Aedes aegypti* from field populations and the susceptible New Orleans strain exposed to LC_50_ and LC_99_ of spiromesifen; Table S11: Lipid content (µg) in females of *Aedes aegypti* from field populations and the susceptible New Orleans strain exposed to LC_50_ and LC_99_ of spiromesifen.
