# Supplementary material for: Repurposing Insecticides for Mosquito Control: Evaluating Spiromesifen, a Lipid Synthesis Inhibitor against Aedes aegypti (L.)
Source: Trop Med Infect Dis. 2024 Aug 18;9(8):184. doi: 10.3390/tropicalmed9080184 (PMC11360630; doi:10.3390/tropicalmed9080184)
Supplement: Supplementary file 1 [file tropicalmed-09-00184-s001.zip › tropicalmed-3153262-supplementary.pdf]

Tropical Medicine and Infectious Disease

MDPI

Supplementary Materials

Table S1. Weight of *Aedes aegypti* larvae (mg) from temephos-resistant populations and the susceptible New Orleans strain exposed to LC_{50} of spiromesifen.

Strain/Population	Control			Treatment		
	24 h	48 h	72 h	24 h	48 h	72 h
New Orleans	3.0618	3.8664	6.0804	3.9424	3.953	5.8589
	2.6324	3.6505	4.9113	2.7648	2.8035	5.4648
	4.4298	3.256	3.275	3.8778	3.4705	4.439
	2.8507	3.516	3.7264	3.0876	3.2448	5.7939
	3.7582	3.79	5.46	2.2896	3.546	4.717
	1.8506	2.4792	3.095	2.7317	3.574	4.1207
	3.4118	3.2056	3.5235	2.9677	3.1158	4.063
	3.1775	3.2494	4.5472	2.9538	2.8396	4.6438
	3.2305	3.8294	4.5405	2.3166	3.3588	5.916
	3.4775	3.4614	5.439	2.3177	3.7244	5.6628
Guadalupe	1.806704	1.918184	2.707179	1.235966	1.92774	1.952806
	1.884999	1.988947	2.131742	1.809084	1.540517	1.787445
	3.851934	1.927691	2.13373	1.994778	1.775595	2.426858
	2.772095	1.909009	2.102152	1.738306	1.765142	2.167311
	3.498872	1.922089	2.108379	1.347055	1.332056	2.230974
	1.953125	1.966093	2.018485	1.3356	1.748903	2.23603
	1.512	1.981829	2.080466	1.278582	1.80542	2.405135
	1.070563	1.951172	2.062509	1.728	1.599609	2.590168
	1.629738	1.937901	1.066039	1.390972	1.15972	2.590168
	1.84363	1.97037	2.034038	1.873954	1.622063	2.613302
Apodaca	1.9804	2.8589	3.0618	1.9424	1.9664	3.953
	1.9113	2.4648	2.6324	1.7648	2.6505	2.8035
	1.275	2.439	2.4298	1.8778	2.256	3.4705
	1.7264	2.7939	2.8507	1.0876	2.516	3.2448
	1.46	2.717	2.7582	1.2896	2.79	3.546
	1.095	2.1207	2.8506	1.7317	3.4792	3.574
	2.5235	2.063	2.4118	1.9677	2.8056	3.1158
	1.5472	2.6438	3.1775	2.9538	2.2494	2.8396
	1.5405	2.916	3.2305	2.3166	2.6294	3.3588

	1.439	2.6628	2.4775	2.3177	2.4614	3.7244
Monterrey	3.442951	4.515456	3.141792	1.685159	4.173281	4.088448
	3.115848	3.229504	4.057463	2.685619	4.019679	4.457463
	2.307949	5.545233	4.331625	3.241792	3.644672	3.511808
	2.515456	3.229504	3.410944	2.019679	2.229504	4.741632
	2.097152	4.826809	3.826809	1	4.301384	4.088448
	2.241792	5.545233	3.211808	2.685619	4.826809	3.924207
	2.352637	5.088448	3.492125	1.753571	3.869893	3.944312
	2.863288	4.615125	3.048625	2.000376	4.229504	3.448625
	2.628072	5.307949	3.051528	3.723875	4.251528	3.146689
	2.628072	4.414875	3.077888	2.146689	4.657463	3.400376

Table S2. Volume of *Aedes aegypti* larvae (mm^3) from temephos-resistant populations and the susceptible New Orleans strain exposed to LC_{50} of spiromesifen.

Strain/Population	Control			Treatment		
	24 h	48 h	72 h	24 h	48 h	72 h
New Orleans	0.91932	1.258814	1.486292	0.847168	0.857425	0.966346
	0.936	0.972901	0.992187	0.819052	0.822688	0.916635
	0.916896	0.923933	1.221419	0.822761	0.874592	0.936481
	0.905965	0.959661	1.4728	0.81522	0.872364	0.946635
	0.857625	0.870661	0.940232	0.823961	0.821484	0.936481
	0.903388	0.993032	1.455889	0.88667	0.866689	0.876725
	0.885174	0.988769	0.991953	0.727775	0.799252	0.872244
	0.939027	0.927998	0.965656	0.891427	0.812784	0.92466
	0.989744	0.994616	1.258649	0.856244	0.821931	0.851273
	0.935404	0.975657	0.998985	0.761184	0.791053	0.867236
Guadalupe	0.851368	1.367631	1.270299	1.367631	0.993039	1.581577
	0.557625	0.951368	1.119679	0.551368	0.551368	1.375
	1.314432	1.625043	1.912673	1.225043	1.771561	1.262144
	0.943032	1.030301	1.946689	1.030301	1.421875	1.331
	0.828509	1.430301	1.707949	1.030301	1.331	1.941192
	1	1.158503	1.871353	0.658503	1	1.295029
	1.314432	1.704969	1.899968	0.704969	0.960375	1.225043
	1.704969	1.984736	2.109968	0.884736	0.771561	1.092727
	1.373248	1.884736	1.953125	0.884736	0.571787	1.958503
	1.092727	1.259712	1.628072	1.259712	1.248091	1.857375
Apodaca	1.295029	1.476523	2.096	1.030301	1.331	1.900376
	1.804357	1.997	2.06523	1.124864	1.176523	1.906624
	1.191016	2.924207	2.960375	1.438976	2.000376	1.307949
	1.771561	2.863288	2.948625	1.515456	2.406104	1.460375
	1.295029	2.571353	2.852264	1.404928	1.096	1.511808

	1.367631	1.8096	1.911808	1.728	1.481544	1.515456
	1.830584	1.911808	1.928072	1.685619	2.352637	1.636056
	1.685159	1.742951	1.942951	1.704969	1.869893	1.581577
	2.000376	2.652264	2.899968	1.571787	1.728	1.042875
	2.685619	2.723875	2.871353	1.753571	1.248091	1.560896
**Monterrey**	2.679184	2.822631	2.76436	2.243796	2.1177	2.101343
	2.542159	2.984853	2.779487	2.263796	2.502846	2.727926
	2.679184	2.853754	2.945221	2.32133	2.383967	2.781067
	2.503792	3.735245	2.129851	1.405497	2.651005	2.345733
	3.551975	3.959752	3.925441	1.428441	3.481097	2.314008
	2.643785	2.906672	2.972787	2.573439	2.106672	2.316905
	2.138155	2.80155	2.744131	1.94644	2.131531	2.375
	2.032296	2.103899	2.264525	1.234434	2.026578	2.789924
	2.90173	2.881577	2.942959	2.915452	2.600451	2.263194
	2.356132	2.401245	2.506726	1.246429	1.926578	2.440432

Table S3. Carbohydrate content in larvae (µg) of *Aedes aegypti* from temephos-resistant populations and the susceptible New Orleans strain exposed to LC_{50} of spiromesifen.

**Strain/Population**	**Control**			**Treatment**		
	**24 h**	**48 h**	**72 h**	**24 h**	**48 h**	**72 h**
**New Orleans**	87	64	82	53	63	62
	85	98	129	52	60	61
	87	85	135	54	64	75
	92	81	127	59	67	73
	84	74	84	56	62	76
	77	71	156	56	64	76
	85	84	131	64	72	66
	81	72	99	55	29	58
	74	101	122	52	46	110
	78	78	102	57	39	118
**Guadalupe**	36	72	110	30	65	143
	55	97	97	37	72	78
	73	77	96	42	60	79
	61	79	133	28	62	90
	47	68	84	21	59	190
	89	111	135	47	69	191
	75	87	135	40	72	88
	58	85	103	28	71	87
	91	73	135	42	104	83
	70	101	142	33	78	97
**Apodaca**	63	74	66	41	91	89

	81	64	62	41	82	68
	61	69	63	34	62	55
	89	90	86	48	81	77
	83	59	53	34	103	98
	83	73	81	44	110	111
	86	93	86	23	57	55
	71	67	67	26	56	51
	71	93	83	47	66	62
	74	94	98	29	74	64
**Monterrey**	67	49	42	34	50	50
	64	50	45	36	45	56
	77	53	61	24	44	54
	64	52	61	33	45	47
	44	46	52	23	45	47
	64	47	58	25	49	51
	53	49	58	32	55	51
	53	50	50	25	51	52
	54	55	43	24	49	47
	58	53	49	25	42	47

Table S4. Lipid content in larvae (µg) of *Aedes aegypti* from temephos-resistant populations and the susceptible New Orleans strain exposed to LC_{50} of spiromesifen.

**Strain/Population**	**Control**			**Treatment**		
	**24 h**	**48 h**	**72 h**	**24 h**	**48 h**	**72 h**
**New Orleans**	80	92	76	80	81	73
	112	100	104	68	72	72
	80	108	120	54	58	72
	77	87	101	65	65	63
	71	92	80	76	76	63
	92	115	124	78	80	70
	96	84	113	69	72	79
	83	95	73	48	50	79
	98	103	133	81	85	99
	84	97	80	81	83	72
**Guadalupe**	79	130	94	76	70	133
	99	122	93	81	62	104
	95	129	93	65	72	102
	79	118	98	60	70	101
	67	149	85	59	87	122
	88	198	102	65	119	143
	83	151	131	72	102	59

	75	143	85	57	94	63
	93	141	116	82	94	82
	82	111	129	91	98	88
**Apodaca**	69	137	42	66	36	43
	88	104	46	66	42	44
	92	92	46	66	39	43
	99	101	45	60	49	45
	59	87	41	52	25	40
	82	97	42	50	28	45
	77	115	36	53	37	26
	55	84	46	54	41	24
	57	88	43	52	28	32
	65	105	50	52	27	33
**Monterrey**	62	40	42	46	21	43
	63	40	46	43	26	44
	58	30	46	48	22	43
	55	48	45	49	24	45
	67	40	41	48	21	40
	50	48	42	45	23	45
	61	42	36	51	36	26
	51	41	46	59	26	24
	51	40	43	47	21	32
	57	53	50	52	20	33

Table S5. Protein content in larvae (µg) of *Aedes aegypti* from temephos-resistant populations and the susceptible New Orleans strain exposed to LC_{50} of spiromesifen.

**Strain/Population**	**Control**			**Treatment**		
	**24 h**	**48 h**	**72 h**	**24 h**	**48 h**	**72 h**
**New Orleans**	175	189	218	212	230	224
	222	197	255	220	250	238
	218	189	259	214	238	214
	211	188	233	216	232	211
	192	188	227	217	221	213
	217	196	271	219	280	215
	208	198	238	221	244	220
	202	194	228	223	223	206
	241	217	271	235	255	252
	208	207	255	229	271	241
**Guadalupe**	260	218	246	268	226	229
	230	283	237	240	229	213
	237	261	263	240	227	243

	261	242	248	246	216	228
	255	234	280	300	239	247
	262	253	262	305	229	252
	300	246	306	219	239	239
	241	229	262	232	235	232
	259	273	252	241	247	246
	295	236	310	262	245	258
**Apodaca**	240	258	179	274	280	163
	221	229	183	243	233	152
	232	239	176	253	236	182
	247	264	185	250	252	181
	235	243	187	281	280	183
	259	254	179	295	311	165
	287	294	184	230	225	162
	243	248	178	224	223	168
	277	271	186	239	229	164
	304	294	186	252	245	173
**Monterrey**	213	216	214	216	211	208
	222	214	201	218	209	209
	223	211	193	188	182	184
	225	215	212	221	201	200
	219	214	215	211	184	209
	221	216	218	218	206	185
	219	215	209	215	208	186
	222	217	212	219	213	200
	219	217	214	217	205	210
	220	159	213	218	185	202

Table S6. Malondialdehyde (μM/ mg of proteins) in larvae of *Aedes aegypti* from temephos-resistant populations and the susceptible New Orleans strain exposed to LC_{50} of spiromesifen.

**Strain/Population**	**Control**			**Treatment**		
	**24 h**	**48 h**	**72 h**	**24 h**	**48 h**	**72 h**
**New Orleans**	0.18888889	0.28219767	0.40513699	0.25909753	0.36003976	0.53581626
	0.19412698	0.1534296	0.33198609	0.22674419	0.3894	0.81677928
	0.19606815	0.24492234	0.30928541	0.23819048	0.33682848	0.64757433
	0.15839568	0.20659472	0.31315193	0.24960317	0.5789302	0.78074866
	0.18338082	0.19050117	0.37728517	0.22592081	0.50073041	0.45571976
	0.18282083	0.22622951	0.32198002	0.23885794	0.58654206	0.84303797
	0.1729979	0.23891925	0.35688358	0.23441882	0.50358974	0.66552083
	0.19642185	0.24718137	0.38188623	0.24718173	0.47125558	0.50250128
	0.17390649	0.22851986	0.48627706	0.21219117	0.40552268	0.75547337

	0.22626932	0.14784946	0.5297486	0.22850934	0.4769419	0.54753086
**Guadalupe**	0.504509631	0.331702	0.540164258	0.76255	0.569046	0.866948569
	0.331245018	0.309375	0.531588579	0.53383131	0.576108	0.748010549
	0.396984602	0.336095	0.511123297	0.455242574	0.525681	0.567001958
	0.618183164	0.463548	0.778139692	0.553774002	0.49232	0.694454678
	0.331827238	0.288654	0.441707486	0.706414108	0.53376	0.846083528
	0.604154941	0.294118	0.787247123	1.143814837	0.673229	0.917421494
	0.565083124	0.35382	0.732393946	0.352145914	0.625091	0.532784683
	0.429411892	0.392139	0.570791203	0.299771843	0.638256	0.499372156
	0.528613595	0.371356	0.809379669	0.365439001	0.622251	0.508509456
	0.641742179	0.398501	0.744714059	0.432855428	0.572138	0.623061149
**Apodaca**	0.660158643	0.373127742	0.622289897	0.650218662	0.604360289	0.966491448
	0.558654368	0.399894594	0.466279596	0.664221376	0.562754831	0.760426156
	0.588728846	0.368210758	0.428803066	0.499681335	0.517367635	0.62187155
	0.742388405	0.253401168	0.879423091	0.615613407	0.603371336	0.737959045
	0.485726962	0.334252427	0.410320365	0.732090323	0.658715834	0.916649944
	0.629880402	0.390147054	0.742702652	0.762816033	0.746360208	1.056003276
	0.587034986	0.4436	0.750039089	0.484999958	0.769080274	0.429661951
	0.547746684	0.431767915	0.555619085	0.46815775	0.730522583	0.371411553
	0.66948601	0.502091557	0.77808423	0.545039759	0.712614444	0.455179832
	0.748068219	0.548262658	0.822950401	0.549084811	0.723490029	0.693998524
**Monterrey**	0.288885529	0.242719	0.297904197	0.239920563	0.450128176	0.291882284
	0.266947378	0.221963	0.294615007	0.237854631	0.383473619	0.302920127
	0.230803417	0.276741	0.338482348	0.221149056	0.392431776	0.316526948
	0.152036133	0.240225	0.269148722	0.210408083	0.399717385	0.309904132
	0.180081832	0.248355	0.280036862	0.208611438	0.383696985	0.308841492
	0.2374512	0.247263	0.259153225	0.220015668	0.46903324	0.347228133
	0.166623945	0.22568	0.322256701	0.177914209	0.372591266	0.316167647
	0.202354756	0.263867	0.28063372	0.209338158	0.436192978	0.324758073
	0.175261714	0.268788	0.279536791	0.139499419	0.358637475	0.26475507
	0.228080591	0.175486	0.271745851	0.229181749	0.360270958	0.328978355

Table S7. Catalase levels (µM/ mg of proteins) in larvae of *Aedes aegypti* from temephos-resistant populations and the susceptible New Orleans strain exposed to LC_{50} of spiromesifen.

**Strain/Population**	**Control**			**Treatment**		
	**24 h**	**48 h**	**72 h**	**24 h**	**48 h**	**72 h**
**New Orleans**	5.02076216	5.44293286	5.65618375	5.3115007	6.02138158	6.23486842
	4.60974178	5.1987395	5.34089636	5.14979592	6.76493711	7.07610063
	4.89563591	5.21830484	5.33675214	5.53387334	6.03388158	6.31940789
	4.88464467	5.3725	5.53808824	5.63278689	6.1252883	7.33574959
	5.28314607	5.91415858	5.0592233	5.47292576	6.03935644	7.18894389

	4.75158537	5.24104585	5.36174785	5.41683453	6.82464342	7.0562599
	4.88666667	5.46549925	5.62488823	5.46215827	6.77291994	7.03139717
	5.18301887	5.65768049	5.80168971	5.76204268	7.87752	7.0512
	4.69341317	4.73719434	4.91776062	4.88471178	6.24878745	6.50727532
	4.69660606	5.45640835	5.75648286	4.94967658	6.50869565	6.74377811
**Guadalupe**	5.43058	4.663537	4.787087	5.143197	6.321973	6.484746
	4.295836	4.392881	5.182062	5.182576	6.849675	5.824547
	4.657834	5.028482	4.824766	5.155321	5.644839	6.467647
	5.024908	4.507134	4.846521	5.391851	4.548941	6.492901
	5.091843	4.74715	4.341757	4.871463	4.748944	5.110772
	4.793274	4.593999	4.7103	5.132871	5.75008	6.814872
	4.90258	4.153012	4.115919	4.897864	5.329216	5.769229
	5.29423	5.015897	4.64313	5.144934	5.080659	5.993546
	4.485236	4.738099	4.904363	4.826957	5.902613	5.896121
	4.95261	4.182733	4.035281	4.762063	5.480688	6.514807
**Apodaca**	5.169815543	4.776170757	6.622849598	4.464151018	6.214410104	8.112656311
	5.427285711	5.236400983	6.552446299	4.94581156	5.13064638	7.910335206
	5.33700949	5.198082911	6.806396465	4.564178311	5.055860586	7.678988992
	4.999151319	4.63197432	6.568585182	4.669686571	5.749446903	7.601142968
	5.062219118	4.925954597	6.336137084	4.281229959	5.250178444	7.707955572
	4.788408897	4.874921682	6.792861331	3.903369749	5.834921432	8.295101494
	4.38829068	4.239873349	6.650481775	5.127170934	6.149029002	8.447722572
	4.998498744	4.873578737	6.69571955	5.21891195	6.372712376	7.491609404
	4.413178182	4.54900676	6.5569859	5.030725341	6.181443165	7.478829109
	4.247582088	4.301427708	6.510397774	4.462208572	5.885220922	7.033436992
**Monterrey**	5.292128785	5.305815244	5.301748664	5.205026479	6.389332979	5.476875006
	5.126040743	5.294679844	5.619502768	5.162967347	7.361373255	5.412480126
	5.060494589	5.391025019	5.8644895	5.945999022	6.896955897	6.192332306
	5.020543429	5.320131151	5.393187231	5.122877695	7.520610444	5.651061952
	5.178038009	5.310831701	5.254098057	5.273469442	7.909882569	5.461438491
	5.127324154	5.264803467	5.179508612	5.183980582	7.454030802	6.159442517
	5.21306377	5.272705939	5.399604331	5.216821896	7.380579262	6.107080279
	5.078318044	5.238533729	5.396209224	5.16514955	7.344197412	5.696150227
	5.173053469	5.207568827	5.300081355	5.166489311	6.416089557	5.473871428
	5.101691653	7.299904196	5.31051262	5.182931816	6.969633614	5.795166683

Table S8. Number of eggs per female after exposure to LC_{50} and LC_{99} of spiromesifen in adult females of *Aedes aegypti* populations and the susceptible New Orleans strain.

Strain/Population	Female	Number of eggs Control	Female	Number of eggs LC_{50}	Female	Number of eggs LC_{99}
New Orleans	1	42	1	0	1	18
	2	24	2	0	2	0
	3	51	3	0	3	0
	4	33	4	0	4	0
	5	17	5	43	5	0
	6	49	6	38	6	23
	7	67	7	21	7	0
	8	94	8	11	8	0
	9	121	9	65	9	34
	10	18	10	38	10	0
	11	56	11	0	11	41
	12	81	12	0	12	27
	13	14	13	56	13	0
	14	39	14	61	14	51
	15	0	15	39	15	0
	16	24	16	0	16	0
	17	12	17	72	17	0
	18	33	18	66	18	24
	19	37	19	0	19	39
	20	86	20	53	20	0
	21	99	21	0	21	0
	22	24	22	40	22	0
	23	36	23	0	23	40
	24	42	24	56	24	0
	25	61	25	42	25	0
	26	0	26	38	26	0
	27	77	27	67	27	0
	28	83	28	0	28	38
	29	141	29	0	29	61
	30	58	30	21	30	0
	31	0	31	38	31	0
	32	63	32	44	32	0
	33	72	33	0	33	0
	34	31	34	0	34	0
	35	17	35	62	35	32
	36	46	36	0	36	0

	37	51	37	71	37	0
	38	53	38	31	38	0
	39	113	39	56		
	40	68	40	18		
	41	41	41	0		
	42	32	42	0		
	43	0	43	0		
	44	60	44	0		
	45	42	45	0		
	46	73	46	46		
	47	18	47	81		
	48	36	48	56		
	49	47	49	0		
	50	92	50	69		
	51	28	51	47		
	52	43	52	35		
	53	107	53	49		
	54	0	54	0		
	55	91	55	0		
	56	24				
	57	38				
	58	62				
	59	19				
	60	42				
	61	67				
	62	122				
	63	92				
	64	22				
	65	47				
	66	81				
	67	0				
	68	0				
	69	56				
	70	71				
	71	32				
	72	24				
	73	51				
	74	21				
	75	66				
	76	39				
	77	28				

	78	52				
	79	34				
	80	40				
	81	59				
	82	27				
	**Female**	**Number of eggs Control**	**Female**	**Number of eggs LC_{50}**	**Female**	**Number of eggs LC_{99}**
	1	0	1	12	1	0
	2	111	2	0	2	0
	3	89	3	0	3	0
	4	46	4	0	4	14
	5	0	5	0	5	0
	6	0	6	55	6	32
	7	77	7	13	7	0
	8	133	8	40	8	0
	9	80	9	32	9	0
	10	22	10	54	10	34
	11	65	11	0	11	48
	12	0	12	0	12	0
	13	43	13	19	13	0
	14	0	14	23	14	0
**Guadalupe**	15	109	15	22	15	0
	16	93	16	38	16	0
	17	0	17	0	17	0
	18	0	18	79	18	37
	19	88	19	43	19	45
	20	67	20	38	20	19
	21	59	21	0	21	0
	22	0	22	0	22	0
	23	38	23	65	23	0
	24	91	24	49	24	0
	25	0	25	37	25	0
	26	167	26	0	26	23
	27	41	27	0	27	41
	28	33	28	0	28	37
	29	0	29	0	29	29
	30	0	30	10	30	11
	31	78	31	90	31	0
	32	73	32	13		

	33	136	33	41		
	34	0	34	0		
	35	0	35	0		
	36	0	36	36		
	37	67	37	11		
	38	30	38	25		
	39	49	39	70		
	40	21	40	35		
	41	69	41	19		
	42	88	42	0		
	43	38	43	0		
	44	91	44	0		
	45	0	45	23		
	46	17	46	38		
	47	54	47	33		
	48	93	48	36		
	49	43	49	0		
	50	68	50	0		
	51	81	51	63		
	52	72	52	57		
	53	66	53	0		
	54	63	54	51		
	55	41	55	50		
	56	27	56	27		
	57	63	57	0		
	58	101	58	0		
	59	67	59	10		
	60	81	60	39		
	61	34	61	14		
	62	53	62	79		
	63	60	63	36		
	64	93	64	41		
	65	19				
	66	24				
	67	83				
	68	77				
	69	30				
	70	69				
	71	51				
	72	34				
	73	80				

	74	112				
	75	67				
	76	89				
	77	46				
	78	32				
	79	98				
	80	43				
	81	55				
	82	80				
	83	29				
	84	74				
	**Female**	**Number of eggs Control**	**Female**	**Number of eggs LC_{50}**	**Female**	**Number of eggs LC_{99}**
	1	65	1	40	1	0
	2	78	2	0	2	0
	3	41	3	65	3	0
	4	101	4	0	4	0
	5	38	5	0	5	0
	6	47	6	0	6	0
	7	66	7	31	7	0
	8	91	8	16	8	0
	9	45	9	27	9	12
	10	0	10	0	10	6
	11	65	11	0	11	0
**Apodaca**	12	78	12	0	12	25
	13	49	13	42	13	0
	14	87	14	0	14	32
	15	95	15	0	15	0
	16	112	16	28	16	0
	17	64	17	56	17	0
	18	43	18	0	18	21
	19	76	19	12	19	0
	20	93	20	48	20	16
	21	37	21	0	21	0
	22	77	22	0	22	13
	23	71	23	29	23	27
	24	65	24	0	24	34
	25	41	25	55	25	43
	26	39	26	17	26	33

	27	0	27	40	27	7
	28	54	28	65		
	29	47	29	34		
	30	76	30	0		
	31	90	31	57		
	32	55	32	19		
	33	87	33	45		
	34	53	34	68		
	35	81	35	0		
	36	39	36	0		
	37	67	37	0		
	38	66	38	70		
	39	34	39	13		
	40	39	40	27		
	41	45	41	0		
	42	79	42	37		
	43	88	43	34		
	44	46	44	22		
	45	99	45	18		
	46	64	46	35		
	47	83	47	0		
	48	55	48	32		
	49	71	49	38		
	50	49	50	0		
	51	70	51	51		
	52	51	52	63		
	53	39	53	15		
	54	44	54	0		
	55	37	55	37		
	56	96	56	41		
	57	77	57	52		
	58	54	58	19		
	59	81				
	60	74				
	61	67				
	62	48				
	63	96				
	64	44				
	65	72				
	66	54				
	67	59				

	68	37				
	69	28				
	70	74				
	71	51				
	72	38				
	73	42				
	74	19				
	75	70				
	76	78				
	77	24				
	78	53				
	79	39				
	80	66				
	81	98				
	82	45				
	83	81				
	84	36				
	85	72				
	86	43				
	87	79				
	88	67				
	**Female**	**Number of eggs Control**	**Female**	**Number of eggs LC_{50}**	**Female**	**Number of eggs LC_{99}**
	1	45	1	0	1	0
	2	67	2	0	2	10
	3	88	3	0	3	7
	4	0	4	0	4	20
	5	100	5	43	5	0
	6	63	6	38	6	19
**Monterrey**	7	78	7	21	7	12
	8	56	8	11	8	31
	9	92	9	65	9	0
	10	72	10	38	10	0
	11	0	11	0	11	0
	12	0	12	0	12	0
	13	84	13	56	13	0
	14	65	14	61	14	41
	15	43	15	39	15	0
	16	89	16	0	16	0

	17	76	17	72	17	0
	18	104	18	66	18	0
	19	0	19	0	19	0
	20	86	20	53	20	0
	21	55	21	0	21	0
	22	84	22	40	22	0
	23	0	23	0		
	24	63	24	56		
	25	126	25	42		
	26	98	26	38		
	27	51	27	67		
	28	77	28	0		
	29	59	29	0		
	30	39	30	21		
	31	78	31	38		
	32	109	32	44		
	33	89	33	0		
	34	43	34	0		
	35	79	35	62		
	36	54	36	0		
	37	131	37	71		
	38	69				
	39	72				
	40	0				
	41	81				
	42	114				
	43	58				
	44	77				
	45	97				
	46	42				
	47	63				
	48	110				
	49	92				
	50	70				
	51	117				
	52	58				
	53	74				
	54	45				
	55	83				
	56	90				
	57	73				

	58	88				
	59	100				
	60	92				
	61	84				
	62	44				
	63	79				
	64	56				
	65	83				
	66	32				
	67	80				
	68	98				
	69	95				
	70	85				
	71	72				
	72	58				
	73	66				
	74	78				
	75	91				
	76	83				
	77	40				
	78	65				
	79	45				
	80	89				
	81	49				
	82	86				
	83	53				
	84	30				
	85	64				
	86	73				

Table S9. Number of eggs hatched per eggs oviposited after exposure to LC_{50} and LC_{99} of spiromesifen in adult females of *Aedes aegypti* populations and the susceptible New Orleans strain.

Strain/Population	Control			LC_{50}			LC_{99}		
	Female	No. of eggs	No. of eggs hatched	Female	No. of eggs	No. of eggs hatched	Female	No. of eggs	No. of eggs hatched
New Orleans	1	42	42	1	0	0	1	18	3
	2	24	24	2	0	0	2	0	0
	3	51	51	3	0	0	3	0	0
	4	33	33	4	0	0	4	0	0
	5	17	17	5	43	10	5	0	0

	6	49	49	6	38	21	6	23	1
	7	67	66	7	21	0	7	0	0
	8	94	94	8	11	0	8	0	0
	9	121	90	9	65	40	9	34	10
	10	18	18	10	38	13	10	0	0
	11	56	45	11	0	0	11	41	7
	12	81	78	12	0	0	12	27	2
	13	14	14	13	56	32	13	0	0
	14	39	39	14	61	27	14	51	19
	15	0	0	15	39	11	15	0	0
	16	24	24	16	0	0	16	0	0
	17	12	12	17	72	41	17	0	0
	18	33	33	18	66	32	18	24	8
	19	37	37	19	0	0	19	39	1
	20	86	86	20	53	20	20	0	0
	21	99	99	21	0	0	21	0	0
	22	24	24	22	40	5	22	0	0
	23	36	36	23	0	0	23	40	4
	24	42	42	24	56	9	24	0	0
	25	61	61	25	42	26	25	0	0
	26	0	0	26	38	4	26	0	0
	27	77	77	27	67	12	27	0	0
	28	83	83	28	0	0	28	38	9
	29	141	121	29	0	0	29	61	2
	30	58	58	30	21	6	30	0	0
	31	0	0	31	38	33	31	0	0
	32	63	63	32	44	7	32	0	0
	33	72	72	33	0	0	33	0	0
	34	31	31	34	0	0	34	0	0
	35	17	17	35	62	14	35	32	17
	36	46	46	36	0	0	36	0	0
	37	51	51	37	71	3	37	0	0
	38	53	53	38	31	24	38	0	0
	39	113	86	39	56	38			
	40	68	68	40	18	2			
	41	41	41	41	0	0			
	42	32	32	42	0	0			
	43	0	0	43	0	0			
	44	60	60	44	0	0			
	45	42	42	45	0	0			
	46	73	73	46	46	15			

	47	18	18	47	81	37			
	48	36	36	48	56	10			
	49	47	47	49	0	0			
	50	92	92	50	69	28			
	51	28	28	51	47	52			
	52	43	43	52	35	3			
	53	107	87	53	49	18			
	54	0	0	54	0	0			
	55	91	57	55	0	0			
	56	24	24						
	57	38	38						
	58	62	62						
	59	19	19						
	60	42	42						
	61	67	67						
	62	122	87						
	63	92	63						
	64	22	22						
	65	47	47						
	66	81	51						
	67	0	0						
	68	0	0						
	69	56	56						
	70	71	49						
	71	32	32						
	72	24	24						
	73	51	51						
	74	21	21						
	75	66	66						
	76	39	39						
	77	28	28						
	78	52	52						
	79	34	34						
	80	40	40						
	81	59	31						
	82	27	27						
	**Control**			**LC_{50}**			**LC_{99}**		
	**Female**	**No. of eggs**	**No. of eggs hatched**	**Female**	**No. of eggs**	**No. of eggs hatched**	**Female**	**No. of eggs**	**No. of eggs hatched**

	1	0	0	1	12	4	1	0	0
	2	100	91	2	0	0	2	0	0
	3	60	45	3	0	0	3	0	0
	4	46	46	4	0	0	4	14	14
	5	0	0	5	0	0	5	0	0
	6	0	0	6	55	12	6	32	7
	7	77	69	7	13	7	7	0	0
	8	133	128	8	40	19	8	0	0
	9	80	65	9	32	7	9	0	0
	10	22	21	10	54	32	10	34	8
	11	65	62	11	0	0	11	48	13
	12	0	0	12	0	0	12	0	0
	13	43	43	13	19	8	13	0	0
	14	0	0	14	23	9	14	0	0
	15	109	100	15	22	17	15	0	0
	16	93	95	16	38	21	16	0	0
	17	0	0	17	0	0	17	0	0
	18	0	0	18	79	40	18	37	0
	19	88	49	19	43	11	19	45	9
	20	67	39	20	38	9	20	19	0
**Guadalupe**	21	59	55	21	0	0	21	0	0
	22	0	0	22	0	0	22	0	0
	23	38	38	23	65	27	23	0	0
	24	91	88	24	49	16	24	0	0
	25	0	0	25	37	8	25	0	0
	26	157	143	26	0	0	26	23	8
	27	41	37	27	0	0	27	41	11
	28	33	31	28	0	0	28	37	16
	29	0	0	29	0	0	29	29	7
	30	0	0	30	10	0	30	11	2
	31	78	59	31	90	41	31	0	0
	32	73	70	32	13	0			
	33	136	136	33	41	27			
	34	0	0	34	0	0			
	35	0	0	35	0	0			
	36	0	0	36	36	0			
	37	67	48	37	11	0			
	38	30	30	38	25	13			
	39	49	47	39	70	28			
	40	21	19	40	35	0			
	41	69	62	41	19	0			

	42	88	68	42	0	0			
	43	38	33	43	0	0			
	44	91	79	44	0	0			
	45	0	0	45	23	0			
	46	17	17	46	38	7			
	47	54	53	47	33	13			
	48	93	84	48	36	19			
	49	43	40	49	0	0			
	50	68	47	50	0	0			
	51	81	69	51	63	48			
	52	72	72	52	57	31			
	53	66	61	53	0	0			
	54	63	59	54	51	22			
	55	41	34	55	50	18			
	56	27	21	56	27	0			
	57	63	46	57	0	0			
	58	77	77	58	0	0			
	59	67	49	59	10	0			
	60	81	78	60	39	2			
	61	34	33	61	14	0			
	62	53	34	62	79	28			
	63	60	59	63	36	22			
	64	93	81	64	41	17			
	65	19	19						
	66	24	24						
	67	66	66						
	68	77	63						
	69	30	30						
	70	69	45						
	71	51	50						
	72	34	31						
	73	80	65						
	74	112	100						
	75	54	54						
	76	89	67						
	77	46	46						
	78	32	31						
	79	98	87						
	80	43	33						
	81	55	46						
	82	80	80						

	83	29	26						
	84	74	65						
	**Control**			**LC_{50}**			**LC_{99}**		
	**Female**	**No. of eggs**	**No. of eggs hatched**	**Female**	**No. of eggs**	**No. of eggs hatched**	**Female**	**No. of eggs**	**No. of eggs hatched**
**Apodaca**	1	65	50	1	40	16	1	0	0
	2	78	63	2	0	0	2	0	0
	3	41	26	3	65	31	3	0	0
	4	101	86	4	0	0	4	0	0
	5	38	28	5	0	0	5	0	0
	6	47	41	6	0	0	6	0	0
	7	66	42	7	31	13	7	0	0
	8	91	48	8	16	0	8	0	0
	9	45	44	9	27	14	9	12	0
	10	0	0	10	0	0	10	6	0
	11	65	56	11	0	0	11	0	0
	12	78	71	12	0	0	12	25	0
	13	49	46	13	42	25	13	0	0
	14	87	84	14	0	0	14	32	9
	15	95	68	15	0	0	15	0	0
	16	112	89	16	28	8	16	0	0
	17	64	51	17	56	32	17	0	0
	18	43	42	18	0	0	18	21	7
	19	76	68	19	12	0	19	0	0
	20	93	82	20	48	13	20	16	3
	21	37	37	21	0	0	21	0	0
	22	77	72	22	0	0	22	13	0
	23	71	55	23	29	0	23	27	18
	24	65	46	24	0	0	24	34	11
	25	41	40	25	55	32	25	43	18
	26	39	33	26	17	0	26	33	10
	27	0	0	27	40	14	27	7	0
	28	54	52	28	65	23			
	29	47	44	29	34	18			
	30	76	74	30	0	0			
	31	90	88	31	57	16			
	32	55	48	32	19	3			
	33	87	79	33	45	27			
	34	53	52	34	68	32			

	35	81	78	35	0	0			
	36	39	32	36	0	0			
	37	67	65	37	0	0			
	38	66	43	38	70	41			
	39	34	33	39	13	0			
	40	39	39	40	27	0			
	41	45	35	41	0	0			
	42	79	61	42	37	11			
	43	88	84	43	34	0			
	44	46	40	44	22	0			
	45	99	80	45	18	0			
	46	64	51	46	35	0			
	47	83	70	47	0	0			
	48	55	43	48	32	8			
	49	71	59	49	38	9			
	50	49	43	50	0	0			
	51	70	70	51	51	22			
	52	51	44	52	63	31			
	53	39	36	53	15	0			
	54	44	43	54	0	0			
	55	37	26	55	37	0			
	56	96	87	56	41	13			
	57	77	63	57	52	23			
	58	54	54	58	19	0			
	59	81	78						
	60	74	74						
	61	67	54						
	62	48	31						
	63	96	76						
	64	44	24						
	65	72	72						
	66	54	43						
	67	59	32						
	68	37	37						
	69	28	22						
	70	74	65						
	71	51	51						
	72	38	38						
	73	42	42						
	74	19	19						
	75	70	67						

	76	78	61						
	77	24	24						
	78	53	43						
	79	39	39						
	80	66	66						
	81	98	92						
	82	45	45						
	83	81	81						
	84	36	36						
	85	72	72						
	86	43	43						
	87	79	79						
	88	67	67						
	**Control**			**LC_{50}**			**LC_{99}**		
	**Female**	**No. of eggs**	**No. of eggs hatched**	**Female**	**No. of eggs**	**No. of eggs hatched**	**Female**	**No. of eggs**	**No. of eggs hatched**
**Monterrey**	1	45	37	1	0	0	1	0	0
	2	67	51	2	0	0	2	10	0
	3	88	71	3	0	0	3	7	0
	4	0	0	4	0	0	4	20	9
	5	100	100	5	43	10	5	0	0
	6	63	54	6	38	21	6	19	5
	7	78	66	7	21	0	7	12	0
	8	56	49	8	11	0	8	31	12
	9	92	90	9	65	40	9	0	0
	10	72	67	10	38	13	10	0	0
	11	0	0	11	0	0	11	0	0
	12	0	0	12	0	0	12	0	0
	13	84	76	13	56	32	13	0	0
	14	65	62	14	61	27	14	41	16
	15	43	36	15	39	11	15	0	0
	16	89	64	16	0	0	16	0	0
	17	76	72	17	72	41	17	0	0
	18	104	104	18	66	32	18	0	0
	19	0	0	19	0	0	19	0	0
	20	86	69	20	53	20	20	0	0
	21	55	48	21	0	0	21	0	0
	22	84	81	22	40	5	22	0	0
	23	0	0	23	0	0			

	24	63	59	24	56	9			
	25	126	112	25	42	26			
	26	98	64	26	38	4			
	27	51	51	27	67	12			
	28	77	63	28	0	0			
	29	59	47	29	0	0			
	30	39	33	30	21	6			
	31	78	78	31	38	33			
	32	109	105	32	44	7			
	33	89	83	33	0	0			
	34	43	43	34	0	0			
	35	79	67	35	62	14			
	36	54	54	36	0	0			
	37	131	128	37	71	3			
	38	69	53						
	39	72	56						
	40	0	0						
	41	81	64						
	42	114	103						
	43	58	52						
	44	77	77						
	45	97	86						
	46	42	34						
	47	63	63						
	48	110	96						
	49	92	84						
	50	70	70						
	51	117	91						
	52	58	53						
	53	74	66						
	54	45	45						
	55	83	83						
	56	90	84						
	57	73	60						
	58	88	75						
	59	100	100						
	60	92	92						
	61	84	79						
	62	44	41						
	63	79	66						
	64	56	56						

	65	83	83						
	66	32	32						
	67	80	74						
	68	98	84						
	69	95	95						
	70	85	78						
	71	72	72						
	72	58	46						
	73	66	51						
	74	78	76						
	75	91	77						
	76	83	83						
	77	40	40						
	78	65	55						
	79	45	45						
	80	89	89						
	81	49	44						
	82	86	62						
	83	53	50						
	84	30	30						
	85	64	58						
	86	73	69						

Table S10. Carbohydrate content (µg) in females of *Aedes aegypti* from field populations and the susceptible New Orleans strain exposed to LC_{50} and LC_{99} of spiromesifen.

**Strain/Population**	**Control**	**LC_{50}**	**LC_{99}**
**New Orleans**	52	53	20
	60	46	28
	53	65	29
	63	52	21
	55	40	29
	57	49	25
	63	41	23
	66	48	35
	67	42	21
	75	45	51
**Guadalupe**	65	27	39
	72	33	28
	64	53	78

	69	43	19
	68	50	34
	67	69	84
	69	70	53
	78	64	65
	83	68	53
	85	69	52
**Apodaca**	65	25	40
	72	46	40
	65	65	26
	82	52	21
	61	68	34
	81	69	60
	81	61	41
	90	28	55
	67	56	41
	87	65	51
**Monterrey**	92	37	40
	84	37	40
	89	40	51
	94	52	46
	67	62	46
	81	69	36
	81	61	41
	90	40	43
	98	44	41
	93	65	51

Table S11. Lipid content (μg) in females of *Aedes aegypti* from field populations and the susceptible New Orleans strain exposed to LC_{50} and LC_{99} of spiromesifen.

Strain/Population	Control	LC_{50}	LC_{99}
**New Orleans**	88	61	66
	90	90	66
	109	86	67
	130	86	45
	108	62	34
	132	85	41
	133	88	41
	109	84	42
	108	86	44

	134	88	40
Guadalupe	92	55	40
	74	55	42
	77	45	65
	80	52	38
	58	46	50
	58	42	64
	105	49	55
	112	66	62
	136	63	25
	151	68	23
Apodaca	107	70	49
	96	67	53
	79	67	60
	75	68	67
	89	65	71
	74	76	68
	84	55	42
	79	57	66
	87	76	67
	72	55	50
Monterrey	97	55	31
	75	48	60
	74	60	81
	111	56	88
	76	55	73
	100	63	26
	111	53	36
	76	45	26
	109	60	38
	99	60	31
